# Learning to grasp and extract affordances: the Integrated Learning of Grasps and Affordances (ILGA) model

**DOI:** 10.1007/s00422-015-0666-2

**Published:** 2015-11-19

**Authors:** James Bonaiuto, Michael A. Arbib

**Affiliations:** Sobell Department of Motor Neuroscience and Movement Disorders, University College London, London, WC1N3BG UK; Neuroscience Program, University of Southern California, Los Angeles, CA 90089-2520 USA; USC Brain Project, University of Southern California, Los Angeles, CA 90089-2520 USA; Computer Science Department, University of Southern California, Los Angeles, CA 90089-2520 USA

**Keywords:** Neural network model, Grasping, Infant development, Affordances

## Abstract

The activity of certain parietal neurons has been interpreted as encoding affordances (directly perceivable opportunities) for grasping. Separate computational models have been developed for infant grasp learning and affordance learning, but no single model has yet combined these processes in a neurobiologically plausible way. We present the Integrated Learning of Grasps and Affordances (ILGA) model that simultaneously learns grasp affordances from visual object features and motor parameters for planning grasps using trial-and-error reinforcement learning. As in the Infant Learning to Grasp Model, we model a stage of infant development prior to the onset of sophisticated visual processing of hand–object relations, but we assume that certain premotor neurons activate neural populations in primary motor cortex that synergistically control different combinations of fingers. The ILGA model is able to extract affordance representations from visual object features, learn motor parameters for generating stable grasps, and generalize its learned representations to novel objects.

**Electronic supplementary material:**

The online version of this article (doi:10.1007/s00422-015-0666-2) contains supplementary material, which is available to authorized users.

## Introduction

The notion of affordances as directly perceivable opportunities for action (Gibson 1966) was used to interpret the activity of certain parietal neurons as encoding affordances for grasping in the FARS model of parieto-frontal interactions in grasping (Fagg and Arbib 1998). However, the FARS model “hard-wires” these affordances, whereas our concern is with the development of these affordances and the grasps they afford. While computational models of infant grasp learning (Oztop et al. 2004) and affordance learning (Oztop et al. 2006) have been developed that work in a staged fashion, there do not exist any models that learn affordance extraction and grasp motor programs simultaneously. This model follows from a suggestion of Arbib et al. (2009) and implements a dual learning system that simultaneously learns both grasp affordances and motor parameters for planning grasps using trial-and-error reinforcement learning. As in the Infant Learning to Grasp Model (ILGM, Oztop et al. 2004), we model a stage of infant development prior to the onset of sophisticated visual processing of hand–object relations, but as in the FARS model (Fagg and Arbib 1998), we assume that certain premotor neurons activate neural populations in primary motor cortex that synergistically controls different combinations of fingers. The issue is to understand how different visual patterns can activate the appropriate subset of these neurons. Specifically, the task of ILGA is to learn (i) “affordances,” representations of object features that indicate where it can be grasped, and (ii) motor parameters that can be used to successfully grasp objects based on these representations.

Newborn infants aim their arm movements toward fixated objects (von Hofsten 1982). These early arm movements have been related to the development of object-directed reaching (Bhat et al. 2005), leading to grasping (Bhat and Galloway 2006), the development of which continues throughout childhood (Kuhtz-Buschbeck et al. 1998). Previous relevant models of infant motor development include Berthier’s (1996), Berthier et al. (2005) and Caligiore et al.’s (2014) models of learning to reach and the ILGM. The thread shared by these models is reinforcement-based learning of intrinsically motivated goal-directed actions based on exploratory movements, or motor babbling: Movements are generated erratically in response to a target and the mechanisms generating the movements are modified via positive reinforcement (Cangelosi and Schlesinger 2013).

Grasping in development seems to increasingly involve visual information in preprogramming the grasp (Lasky 1977; Lockman et al. 1984; Von Hofsten and Ronnqvist 1988; Clifton et al. 1993; Newell et al. 1993; Witherington 2005). In ILGM, the affordance extraction module only represented the presence, position, or orientation of an object. All fingers were extended in the initial “preshape” portion of the grasp to a maximal aperture. Initially, the enclosure was triggered by the palmar reflex upon object contact. However, each time the result of reflex grasping provided a stable grasp, this grasp was reinforced, and over time, a repertoire developed of situations in which a stable grasp could be elicited—including the appropriate prepositioning of the hand to a position from which the final approach of hand to object could be made—without relying on the happenstance of reflex grasping. However, the model did not learn the affordances of objects and a fortiori could not exploit them by preshaping appropriately during the reach to grasp. This shortcoming motivates the Integrated Learning of Grasps and Affordances (ILGA) model which simulates the way in which affordance extraction and grasp specification may be adapted simultaneously. It models the developmental transition to hand preshape based on visual information (Von Hofsten and Ronnqvist 1988; Schettino et al. 2003; Witherington 2005) and utilizes the “virtual finger hypothesis” for hand control during grasping. The virtual finger hypothesis states that grasping involves the assignment of real fingers to the so-called virtual fingers (VFs) or force applicators (Arbib et al. 1985). For example in a power grasp, one virtual finger might be the thumb and the other might be the palm while a precision pinch might oppose the thumb to a virtual finger comprising one or more of the other fingers. The task of grasping is then to preshape the hand according to the selected virtual fingers and the size of the object and bring the opposition axis of the virtual fingers into alignment with the selected object surface opposition axis (grasp affordance). Experimental evidence consistent with this hypothesis, also known as hierarchical control of prehension synergies, has been found (Smeets and Brenner 2001; Zatsiorsky and Latash 2004; Winges and Santello 2005). These studies suggest that grasp force planning occurs in two hierarchical levels, with virtual finger force planning occurring first and then planning at the level of individual fingers, which provides a common input to motor neurons in various finger muscles (Zatsiorsky and Latash 2004; Winges and Santello 2005). However, note that it has also been suggested that prehension synergies are not well represented in motor cortex and that the role of motor cortex is to modulate subcortically represented synergies in order to allow individual control of the fingers (Mollazadeh et al. 2014).

In the FARS model, ventral premotor region F5 contained populations of neurons *prewired *for each grasp type (precision pinch, power grasp, etc.). Within each population, subpopulations were selective for each phase of the grasp. In the behavioral protocol used in the FARS simulations, this included Set, Extension, Flexion, Hold, and Release; however, only Set, Extension, and Flexion are considered here. Neurons in each Set subpopulation excited neurons in the same subpopulation and inhibited those neurons in Set subpopulations selective for other grasps. This connectivity implemented a winner-take-all dynamic that selected which grasp to perform based on affordance input from the anterior intraparietal area AIP. The grasp was controlled by activating F5 populations that encoded each phase of the grasp (although some neurons might be active across more than one consecutive phase). Feedback projections from F5 neurons to AIP modulated its activity according to the grasp phase.

In FARS, the secondary somatosensory cortex (SII) detected when the hand aperture reached the predicted maximal value for the planned grasp and triggered grasp enclosure by activating the appropriate Flexion subpopulation in F5. ILGM included detection of object contact with the palm in SI that automatically triggered enclosure. These represent feedforward and naïve feedback strategies, respectively. However, a more sophisticated feedback strategy might be used where the detection of the hand approaching the object is used to trigger the grasp enclosure. This is still a feedback strategy in that the relative position of hand and target object is used to trigger the enclosure, but does so before the hand contacts the object as in the naïve feedback strategy.

Before delving further into the anatomy, it is important to note that we are conflating neurophysiological and neuroanatomical data from macaques with behavioral and lesion data from humans. Thus, the areas that ILGA simulates are primarily rooted in macaque neuroanatomy. However, it is widely accepted that the basic mechanisms of the reach and grasp are conserved across monkeys, apes, and humans, and thus the ILGA model, like the FARS model, is assumed to apply equally well to the macaque and (with suitable alignment of homologous brain regions) for apes and humans. The FARS model made an important distinction between the processing of visual input via the dorsal and ventral streams. The dorsal stream was modeled as extracting the affordances of the attended object, whereas the ventral stream could extract the identity of the object and could thus supply data to prefrontal cortex (PFC) to use in determining which of the affordances would best meet current task demands and working memory. The division of labor can be exemplified by having the ventral stream recognize a mug, recall that it still contains coffee, deciding to drink it, and thus selecting the handle for grasping; but it is up to the dorsal stream to process shape information to extract the affordance of the handle and the motor parameters of the grasp appropriate to that affordance.

Since the distinction was first made between the roles of dorsal and ventral visual streams in grasping (Goodale and Milner 1992; Jeannerod et al. 1994), the dorsal stream has been further subdivided into the dorsal-medial and dorsal-ventral streams (Rizzolatti and Matelli 2003). It has been suggested that the dorsal-medial stream, involving superior parietal and intraparietal regions and the dorsal premotor cortex, controls reaching, while the dorsal-ventral stream, including inferior parietal and intraparietal regions and the ventral premotor cortex, controls grasping (Jeannerod et al. 1995; Wise et al. 1997). The main regions of the dorsal-medial stream seem to include the medial intraparietal area (MIP) and area V6A in the parietal cortex, and area F2 in the dorsal premotor cortex. The spatial dimensions of potential targets such as direction and distance are likely processed independently in parallel (Battaglia-Mayer et al. 2003). In support of this idea, direction and distance reach errors dissociate (Soechting and Flanders 1989; Gordon et al. 1994) and distance information decays faster than direction information in working memory (McIntyre et al. 1998). Our model therefore dissociates the representation of direction and distance in order to reduce the dimensionality of spatial representations to be associated with motor parameter values. However, we note data suggesting that many neurons are modulated by a combination of both variables (Fu et al. 1993; Messier and Kalaska 2000).

**Fig. 1:**
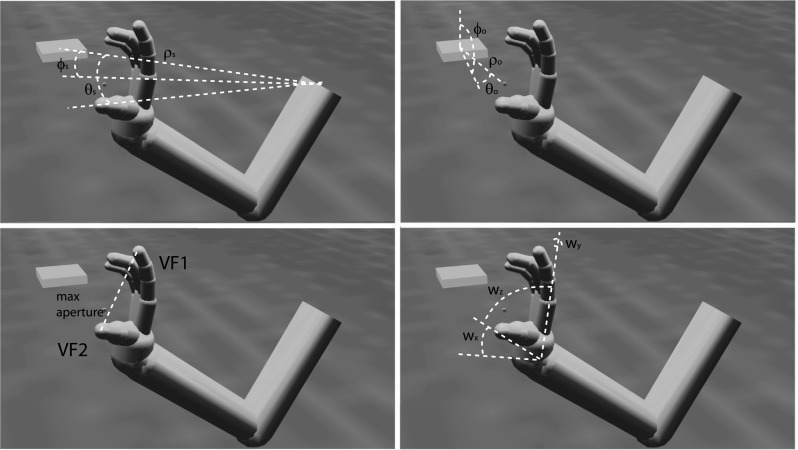
Reach and grasp parameters encoded by the premotor cortex. The *small cube* denotes the planned reach offset point, and the larger rectangular prism is the object to be grasped. As explained later, area F2 encodes the shoulder-centered object position, ${\varphi }_\mathrm{s}$, ${\theta }_\mathrm{s}$, ${\rho }_\mathrm{s}$, F7 encodes the object-centered reach offset, ${\varphi }_\mathrm{o}$, ${\theta }_\mathrm{o}$, ${\rho }_\mathrm{o}$, F5 encodes the VF combination and maximum aperture used for the grasp, and F2/F5 encodes the wrist orientation $\hbox {wr}_{x}$, $\hbox {wr}_{y}$, $\hbox {wr}_{z}$

ILGA learns to perform successful grasps by developing mappings between visual object features and various parameters used to control the arm and hand (Fig. 1). The grasp is determined by the selection of virtual fingers, VF1 and VF2, as well as the maximum aperture. While studies of parietal representation of object location test the encoding of the center of a visual target, reach-to-grasp movements direct the wrist to some region offset from this point so that the hand may contact the object’s affordances appropriately. Similar to the ILGM, ILGA learns to select an object-centered reach offset in spherical coordinates, $\varphi _\mathrm{o},\, \theta _\mathrm{o},\, \rho _\mathrm{o}$. This vector is combined with a shoulder-centered representation of the object center, $\varphi _\mathrm{s},\, \theta _\mathrm{s},\, \rho _\mathrm{s}$, in order to compute a reach target. Once the wrist reaches this point, the reach controller aims for the center of the object, ensuring that the hand approaches the object from the chosen direction. Along with affordance information, the selected offset and grasp type influence the selection of the wrist orientation, $\text {wr}_{x},\, \text {wr}_{y}$, and $\text {wr}_{z}$.

## Methods

The simulation environment is composed of

1. the Neural Simulation Language (NSL) simulator interfaced with the Open Dynamics Engine (ODE, http://www.ode.org) for physics simulation and Java3D (http://java3d.java.net) for visualization,
2. a new model of the primate arm and hand, and
3. the implementation of the ILGA model in NSL.

### The simulation environment

In order to embody models in simulated environments, the Java version of the Neural Simulation Language (NSL, Weitzenfeld et al. 2002) simulator has been extended to include 3D graphics functionality and a physics engine. Utilities were developed to create a simulated 3D environment and embed bodies in this environment with limbs connected by hinge, universal, or ball joints. The 3D graphics function, physics engine, and 3D simulation utilities allow NSL models to control bodies in a simulated 3D world and to receive virtual sensory input from the environment.

The simulated 3D world uses Java3D to maintain a scene graph—a data structure commonly used in computer games to represent the spatial configuration of objects in a scene. Geometric transformations and compound objects are efficiently handled by associating transformation matrices with graph nodes. These matrices can be transformed in order to move an object and all of its child objects (e.g., moving the elbow moves the arm and the hand).

The Open Dynamics Engine (ODE) is used for the physics simulation. ODE is an open source library for simulation of rigid body physics. It contains several joint types and performs collision detection (and contact force application) with friction. When the engine is initialized, NSL maintains a coupling between it and the Java3D representation. At each time step, the physics engine is polled for the position and orientation of the object which is used to update the object’s position and orientation in the Java3D scene graph. Forces can be applied to objects in the scene and torque to objects connected with joints.

### Modeling the primate arm and hand

**Fig. 2:**
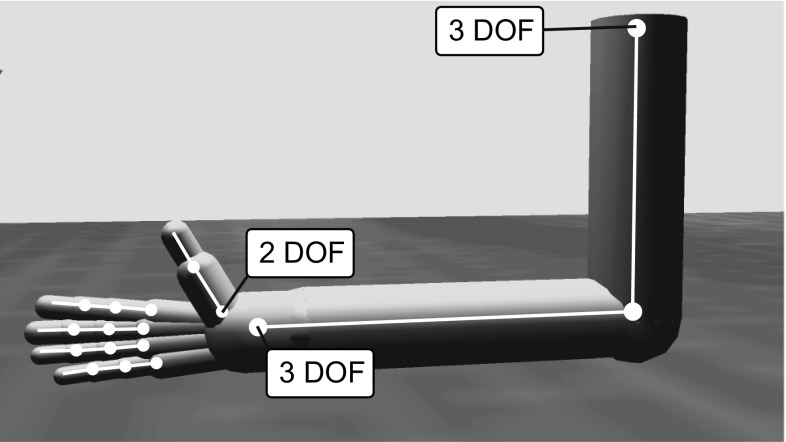
Unless specified, each joint has 1 DOF. The simulated arm/hand has a total of 22 DOFs

The arm and hand generate forces on the object via the ODE, which in turn is used to estimate grasp stability. To facilitate translation of ILGA to a robotic implementation (see Sect. 4), we chose to model the arm and hand as realistically as possible within the confines of our simulation engine. We implemented a 22-degree of freedom (DOF) arm/hand model using limb proportions based on those for a 7.5-kg monkey (Chan and Moran 2006). The arm has a ball joint at the shoulder with 3 DOFs, a 1-DOF hinge joint at the elbow, and a 3-DOF ball joint at the wrist (Fig. 2). The fingers each have three joints with 1 DOF for the metacarpophalangeal, proximal interphalangeal, and distal interphalangeal joints, while the thumb has one 2-DOF joint at its base (simplifying the carpometacarpal joint) and a 1-DOF metacarpophalangeal joint.

The forward kinematics of the arm is described by a matrix, known as the Jacobian matrix, which transforms joint angles of the shoulder and elbow into resulting wrist positions. The inversion of the Jacobian can then be used to determine required joint angles for a desired wrist position. The Jacobian matrix for the arm with upper arm length $l_{1}$ and forearm length $l_{2}$ is given by:

$$\begin{aligned} \mathbf{J}= & {} \left[ {{\begin{array}{ll} {-\left( {\left( {{s}_{3} {c}_{1} {-c}_{3} {s}_{2} {s}_{1} } \right) {c}_{4} -\left( {{-s}_{3} {s}_{1} {-c}_{3} {s}_{2} {c}_{1} } \right) {s}_{4} } \right) {l}_{2} -\left( {{s}_{3} {s}_{1} {+c}_{3} {s}_{2} {c}_{1} } \right) {l}_{1} }&{} {-\left( {\left( {{c}_{3} {c}_{2} {c}_{1} } \right) {c}_{4} -\left( {{-c}_{3} {c}_{2} {s}_{1} } \right) {s}_{4} } \right) {l}_{2} {-c}_{3} {c}_{2} {s}_{1} {l}_{1} } \\ {-\left( {\left( {{-c}_{3} {c}_{1} {-s}_{3} {s}_{2} {s}_{1} } \right) {c}_{4} {-(c}_{3} {s}_{1} {-s}_{3} {s}_{2} {c}_{1} {)s}_{4} } \right) {l}_{2} -\left( {{-c}_{3} {s}_{1} {+s}_{3} {s}_{2} {c}_{1} } \right) {l}_{1} }&{} {-\left( {\left( {{s}_{3} {c}_{2} {c}_{1} } \right) {c}_{4} -\left( {{-s}_{3} {c}_{2} {s}_{1} } \right) {s}_{4} } \right) {l}_{2} {-s}_{3} {c}_{2} {s}_{1} {l}_{1} } \\ {-\left( {\left( {{-c}_{2} {s}_{1} } \right) {c}_{4} -\left( {{-c}_{2} {c}_{1} } \right) {s}_{4} } \right) {l}_{2} {-c}_{2} {c}_{1} {l}_{1} }&{} {-\left( {\left( {{-s}_{2} {c}_{1} } \right) {c}_{4} -\left( {{s}_{2} {s}_{1} } \right) {s}_{4} } \right) {l}_{2} {+s}_{2} {s}_{1} {l}_{1} } \\ \end{array} }} \right. \\&\left. {{\begin{array}{ll} {-\left( {\left( {{c}_{3} {s}_{1} {-s}_{3} {s}_{2} {c}_{1} } \right) {c}_{4} -\left( {{c}_{3} {c}_{1} {+s}_{3} {s}_{2} {s}_{1} } \right) {s}_{4} } \right) {l}_{2} -\left( {{-c}_{3} {c}_{1} {-s}_{3} {s}_{2} {s}_{1} } \right) {l}_{1} }&{} {-\left( {-\left( {{s}_{3} {s}_{1} {+c}_{3} {s}_{2} {c}_{1} } \right) {s}_{4} -\left( {{s}_{3} {c}_{1} {-c}_{3} {s}_{2} {s}_{1} } \right) {c}_{4} } \right) {l}_{2} } \\ {-\left( {\left( {{s}_{3} {s}_{1} {+c}_{3} {s}_{2} {c}_{1} } \right) {c}_{4} -\left( {{s}_{3} {c}_{1} {-c}_{3} {s}_{2} {s}_{1} } \right) {s}_{4} } \right) {l}_{2} -\left( {{-s}_{3} {c}_{1} {+c}_{3} {s}_{2} {s}_{1} } \right) {l}_{1} }&{} {-\left( {-\left( {{c}_{3} {s}_{1} {+s}_{3} {s}_{2} {c}_{1} } \right) {s}_{4} -\left( {{-c}_{3} {c}_{1} {-s}_{3} {s}_{2} {s}_{1} } \right) {c}_{4} } \right) {l}_{2} } \\ 0&{} {-\left( {-\left( {{c}_{2} {c}_{1} } \right) {s}_{4} -\left( {{-c}_{2} {s}_{1} } \right) {c}_{4} } \right) {l}_{2} } \\ \end{array} }} \right] \end{aligned}$$

where ${\sin }(\theta _{1})$ and $\hbox {cos}(\theta _{1})$ are abbreviated as $s_{1}$ and $c_{1},\, \hbox {sin}(\theta _{2})$ and $\hbox {cos}(\theta _{2})$ as $s_{2}$ and $c_{2},\, \hbox {sin}(\theta _{3})$ and $\hbox {cos}(\theta _{3})$ as $s_{3}$ and $c_{3}$, and $\hbox {sin}(\theta _{4})$ and $\hbox {cos}(\theta _{4})$ as $s_{4}$ and $c_{4}$. The angle $\theta _{1}$ is the angle of the shoulder in the *x*-axis, $\theta _{2}$ is the shoulder angle in the *y*-axis, $\theta _{3}$ is the shoulder angle in the *z*-axis, and $\theta _{4}$ is the elbow angle.

Proportional-derivative (PD) controllers are used to control each DOF of the arm and hand. A PD controller adjusts a variable until it reaches a target value and includes gain and damping parameters which can be adjusted to produce fast and smooth trajectories. In ILGA, each PD controller applies torque $\tau$ at time *t* to its controlled joint with angle $\theta$ in order to reach to a desired value $\hat{{\theta }}$:

$$\begin{aligned} \tau (t)=p\left( {\hat{{\theta }}-\theta (t)} \right) +d\dot{\theta }(t) \end{aligned}$$

where *p* is the gain and *d* is a damping parameter (see Online Resource 1, Table 1 for parameter values).

**Fig. 3:**
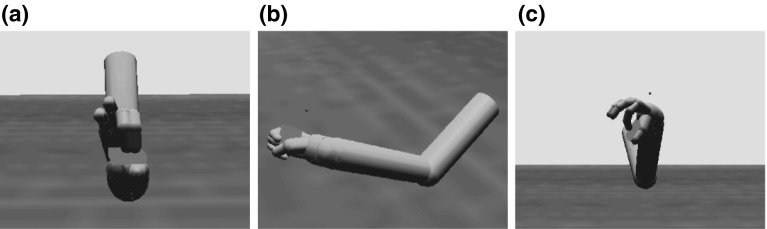
Examples of the model successfully grasping the same object with different types of grasps. **a** Tripod, **b** power, **c** precision pinch

The torque from the joint controllers is applied to the arm and hand segments in the physics model, which updates the position of all of the rigid bodies in the simulated world including the object. Stable grasps are evaluated by monitoring contact points between the fingers and the object in order to provide the reinforcement signal. Figure 3 provides three examples of the arm and hand positioned to grasp an object. In each case, we show a configuration that ILGA can achieve after it has learned to extract affordances from an object and pair them with appropriate grasps.

### Integrated Learning of Grasping and Affordances

**Fig. 4:**
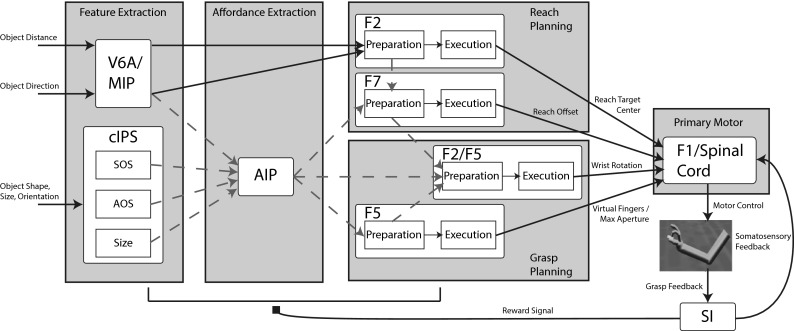
An overview of the ILGA model. Connections modifiable by reinforcement learning are shown in dashed lines. The parietal regions MIP and V6A provide the premotor region F2 with object position information to plan the reach. V6A, MIP, and the cIPS populations project to AIP, which projects to the signal-related populations of the other premotor regions. Each premotor region selects a value for the parameter it encodes and projects to the primary motor region F1 which controls the movement. The reach planning is performed by area F2 which represents the center of the target object and F7 which selects the reach offset. Grasp planning is performed by area F5 which selects the virtual fingers and maximum grasp aperture and F2/F5 which selects the wrist rotation. Grasp feedback is returned to somatosensory area S1 which provides the reinforcement signal to the model and somatosensory feedback to F1. Preparation-related premotor populations plan the reach and excite corresponding execution-related premotor populations. Each execution-related premotor population additionally receives tonic inhibitory inhibit (not shown) that is released when a go signal is detected, triggering the performance of the reach and grasp

The main modules in ILGA are the Feature and Affordance Extraction, Reach and Grasp Planning, and Primary Motor modules (Fig. 4). The Feature Extraction module represents the metric features of graspable objects such as their location, orientation, shape, and oriented surfaces. This information is passed on to the Affordance Extraction module which learns to combine this information into representations of affordances for grasping the object. These representations are used by the Reach and Grasp Planning modules to select motor parameters for reaching to and grasping the object. The Primary Motor module decodes these parameters and controls the reach and grasp movements. Grasp success is monitored by the primary somatosensory (S1) module and is used to generate a reinforcement signal which modifies the connections between the Feature and Affordance Extraction modules and those between the Affordance Extraction and Reach and Grasp Planning modules.

The Affordance Extraction module receives basic object information such as location, size, shape, and orientation in the form of population codes from the Feature Extraction module. The Affordance Extraction module contains a self-organizing feature map (SOM) with its learning rate modulated by a global reinforcement signal. SOMs use unsupervised learning (although the version used in ILGA is semi-supervised since the learning rate is modulated by a reinforcement signal) to map a high-dimensional vector space onto a lower-dimensional space. The resulting map preserves topological relationships between vectors (i.e., similar vectors in the high-dimensional input space are mapped onto nearby vectors in the low-dimensional output space, albeit with some possible discontinuities) and identifies dimensions in the input space with the highest variance. The preservation of topological relationships allows the network to generalize to objects it has never seen before but nonetheless elicit activation patterns overlapping those generated by objects in the training set. They are similar to other dimensionality reduction methods such as multidimensional scaling (MDS) and principal components analysis (PCA, Yin 2008), and it has been argued that a similar mechanism organizes representations of high-dimensional spaces in the cerebral cortex (Durbin and Mitchison 1990). Our addition of modulation by a reinforcement signal causes the network to preferentially represent input vectors that are used to generate grasps which result in a positive reward signal by effectively increasing the variance in these regions of input space, a method comparable to the reinforcement-driven dimensionality reduction model (Bar-Gad et al. 2003). The result of training is a network that can extract combinations of object features that afford successful grasps (affordances).

Neurons in the Affordance Extraction module activate dynamic neural fields (DNFs, Amari and Arbib 1977; Erlhagen and Schoner 2002) in the Reach and Grasp Planning modules that select parameters for grasping such as reach offset, wrist rotation, grasp type, and maximum aperture. DNFs utilize cooperation and competition between neurons depending on their preferred stimulus values. In their most basic form, DNFs implement a winner-take-all (WTA) process, resulting in a population code centered on the cell with the highest mean input. In ILGA, we use one-, two-, and three-dimensional DNFs as WTA networks to select grasp parameters. Each neuron in every DNF has a preferred stimulus value for each dimension of the encoded parameter. In general, the preferred values of each unit could be set arbitrarily, but we set them in a regular fashion such that the population defines a grid in stimulus space (one-, two-, or three-dimensional depending on the dimensionality of the DNF).

Below, we specify the equations used by the Primary Motor module (F1/Spinal Cord in Fig. 4) to decode the activities of the Reach and Grasp Planning module DNFs to obtain the values of various parameters used to plan the reach and grasp movement. Note that the Primary Motor module is *not *modeled as a neural network, but supplies the inputs needed by the physics simulator to apply torques to the joints of the hand and arm. Due to noise in each layer, a random grasp plan can be generated with a small probability, which encourages exploration of the parameter space. Noise levels were set empirically to optimize the trade-off between exploration and exploitation.

Reinforcement learning modifies the connections to and from Affordance Extraction module, resulting in (i) affordances: representations in of combinations of object features relevant for grasping, and (ii) grasp plans: connection weights between the Affordance Extraction and Reach and Grasp Planning modules that bias selection of appropriate grasp motor parameters. The realistic physics simulator we use allows evaluation of grasp stability based on whether or not the grasp can be maintained. Grasps which do not apply forces to appropriate contact points on the object will cause the object to rotate and slip from the hand’s grasp. Positive reinforcement is given by the realization of a stable grasp of the target object, and negative reinforcement is given for grasps that do not contact the object or are unstable enough to allow the object to slip from the hand. The result is that Affordance Extraction neurons are shaped to provide “better” affordance input for the Reach and Grasp Planning module, which in turn expands the repertoire of grasp actions providing more data points for Affordance Extraction learning. Eventually, a stable state is reached when affordance representations are nearly static and grasp performance reaches an upper limit. When this dual learning system stabilizes, the model is endowed with a set of affordance extraction and robust grasp planning mechanisms.

### Reinforcement

Rather than relying on basic extrinsic reward signals from primary reinforcers, it has been suggested that hierarchies of skills could be learned based on intrinsic reward signals that reinforce unexpected salient events (Chentanez et al. 2004). Infants as young as 3–4 months old have experience with objects being placed in their grasp and seem intrinsically motivated to reach for and grasp objects themselves (Thelen et al. 1993). The ILGM thus posited an intrinsic “joy of grasping” as the reward stimulus generated from sensory feedback resulting from the stable grasp of an object. We use the same signal to train the connection weights in this model using reinforcement learning (Sutton and Barto 1998). However, this model uses a more realistic physics simulator than the ILGM, taking into account not only kinematics but also dynamics. This makes motor control a much more difficult task, but simplifies grasp stability evaluation (see below). Another consequence is that since the object can be moved, hand–object collision can knock the object out of reach, making successful grasps much less likely to occur by chance during trial-and-error learning. In order to increase the probability of successful grasps, we pretrain the connection weights that determine the direction of hand approach to the object and the wrist orientation using a more basic reinforcement signal, what may be called the “joy of palm contact.” After pretraining these connection weights, the majority of attempted grasps make at least transient palm contact with the object, increasing the number of stable grasps during the next stage of training. We therefore model palm contact as an unexpected and intrinsically rewarding salient event during early training. As the infant becomes more proficient in reaching for and orienting their hand toward the object, we suggest that palm contact is less novel and therefore less intrinsically rewarding. At this stage, stable grasps are the primary intrinsically rewarding events.

The ILGM used a kinematic simulator that did not handle dynamics and therefore had to use an ad hoc scheme to estimate grasp stability. Since we use a physics simulator that handles rigid body dynamics including friction, grasp stability evaluation is more direct. We ran all simulations reported here with gravity turned off and the object suspended at various locations in order to simplify control of the arm and hand. Even without gravity, the physics simulation will cause the object to slip from the hand’s grasp if the grasp is unstable. The simulator informs the model of the list of contact points between the hand and the object. If two contact points are achieved that define an opposition axis (a vector connecting them in space) that passes through the object, and these contact points are maintained for 2 seconds of simulation time, the grasp is declared successful. Note that none of the other modeled regions have any notion of contact points—the grasp is planned and controlled in an open loop manner. However, contact point feedback could be used to learn internal models for feedback-based grasp control (see Sect. 4).

### Primary motor module: reach and grasp generation

With the Primary Motor module (F1 and the Spinal Cord in Fig. 4), we leave the domain of neural networks: The module decodes the motor parameters for the reach and grasp from the activities of the premotor populations and directs the actual movement by setting the joint angle targets of the PD controllers for each DOF. While future versions of the model may implement this module in a more neurobiologically plausible way, we chose to implement it using techniques from robotics in order to simplify the neural components of the model and focus on parietal and premotor neural activity. Although non-neural, the Primary Motor module serves to achieve the planned grasp end state in order to evaluate the success of the chosen parameter values in producing a stable grasp.

**Fig. 5:**
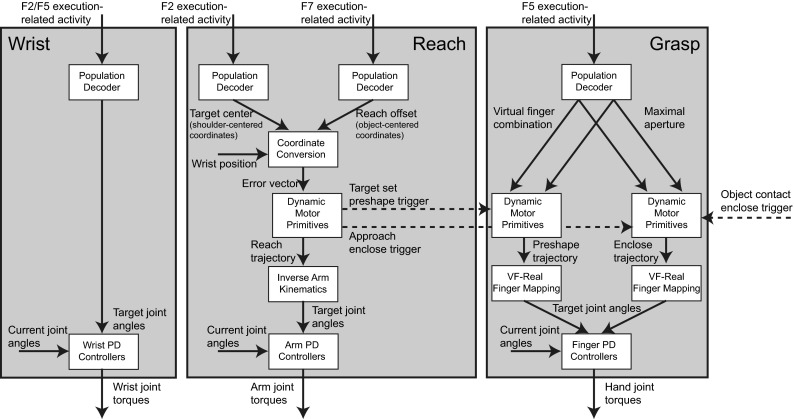
Wrist, reach, and grasp motor controllers. Each uses population decoders to decode reach and grasp parameter values from premotor inputs and set joint angle targets for PD controllers which move the limbs by applying torque to the joints. The reach motor controller combines the shoulder-centered object position, object-centered reach offset, and current wrist position to compute a wrist error vector. The error vector is used to set goal values for dynamic movement primitives, which generate a reach trajectory for the wrist. An inverse arm kinematics module computes target joint angles for each target wrist position. The grasp motor controller contains dynamic movement primitives for the preshape and enclose phases that are triggered by reach and tactile events. These dynamic movement primitives generate normalized trajectories for each virtual finger that are converted into target joint angles by $\hbox {VF}\,\rightarrow \,\text {real}$ finger mapping modules

The wrist rotation, reach, and grasp components of the movement are handled by separate controllers (Fig. 5). The reach and grasp components are coupled by starting the preshape phase of the grasp once the reach target has been determined, and triggering the enclose phase once the hand reaches a certain distance from the object or achieves palm contact. Palm or inner thumb contact is also used as a signal to stop the reach controller at the current wrist position. Each controller decodes the input it receives from premotor execution-related populations, transforms this input along with some proprioceptive or tactile signals, and sets joint angle targets for PD controllers that apply torque to each joint.

Each neuron in every DNF has a preferred stimulus value for each dimension $(\hat{{x}},\,\hat{{y}},\,\hat{{z}})$ of the parameter it encodes. Reach and grasp parameter values were decoded from the activity of each DNF using the center-of-mass technique (Wu et al. 2002). Since noise can greatly bias this form of decoding in small populations, we only include the activities of neurons that pass a threshold, $\xi$ (set to 0.01 in these simulations). For a one-dimensional DNF, the encoded value, *x*, was estimated as:

$$\begin{aligned} x=\frac{\sum _i {\left( {\mathbf{f}\left( i \right) {\hat{\mathbf{x}}}\left( i \right) } \right) } }{\sum _i {\mathbf{f}\left( i \right) } } \end{aligned}$$

where the sums are over all neurons *i* with an activation greater than or equal to the threshold, $\xi$. Similarly, the encoded *x*, *y* value was estimated from a two-dimensional DNF as:

$$\begin{aligned} x= & {} \frac{\sum _i {\left( {\sum _j {\left( {\mathbf{f}\left( {i,j} \right) {\hat{\mathbf{x}}}\left( i \right) } \right) } } \right) } }{\sum _i {\left( {\sum _j {\mathbf{f}\left( {i,j} \right) } } \right) } },\\ y= & {} \frac{\sum _j {\left( {\sum _j {\left( {\mathbf{f}\left( {i,j} \right) {\hat{\mathbf{y}}}\left( j \right) } \right) } } \right) } }{\sum _i {\left( {\sum _j {\mathbf{f}\left( {i,j} \right) } } \right) } } \end{aligned}$$

and the $x,\, y,\, z$ value encoded by a three-dimensional DNF as:

$$\begin{aligned} x= & {} \frac{\sum _i {\left( {\sum _j {\left( {\sum _k {\left( {\mathbf{f}\left( {i,j,k} \right) {\hat{\mathbf{x}}}\left( i \right) } \right) } } \right) } } \right) } }{\sum _i {\left( {\sum _j {\left( {\sum _k {\mathbf{f}\left( {i,j,k} \right) } } \right) } } \right) } },\\ y= & {} \frac{\sum _i {\left( {\sum _j {\left( {\sum _k {\left( {\mathbf{f}\left( {i,j,k} \right) {\hat{\mathbf{y}}}\left( i \right) } \right) } } \right) } } \right) } }{\sum _i {\left( {\sum _j {\left( {\sum _k {\mathbf{f}\left( {i,j,k} \right) } } \right) } } \right) } },\nonumber \\ z= & {} \frac{\sum _i {\left( {\sum _j {\left( {\sum _k {\left( {\mathbf{f}\left( {i,j,k} \right) {\hat{\mathbf{z}}}\left( i \right) } \right) } } \right) } } \right) } }{\sum _i {\left( {\sum _j {\left( {\sum _k {\mathbf{f}\left( {i,j,k} \right) } } \right) } } \right) } } \end{aligned}$$

The simplest controller, the *wrist rotation controller*, decodes the target wrist angles from its premotor input and passes them as target angles to the wrist PD controllers. The *reach controller* combines the output of F2 encoding the center of the object, with the output of F7 encoding the object-centered reach offset into a wrist-centered error vector defining the initial target of the reach. The reach controller couples a trajectory planning mechanism (dynamic movement primitives, DMPs, Ijspeert et al. 2002) with an inverse arm kinematics module. Given a reach target location, the reach planner uses DMPs to generate a trajectory of desired wrist locations to reach it starting from the current wrist position. DMPs can generate arbitrary trajectories and dynamically adapt to new goals. They are defined by the following differential equation:

$$\begin{aligned} \dot{v}= & {} \textit{uK}\left( {\frac{\sum _i {\psi _i \left( u \right) \left( {c_i +x_0 } \right) } }{\sum _i {\psi _i \left( u \right) } }-x} \right) +\left( {1-u} \right) K\left( {g-x} \right) \\&-\,\textit{Dv} \end{aligned}$$

where *x* is the current value of the controlled variable (the position of the wrist in this case), $x_{0}$ is the initial value (the starting position of the wrist), *v* is the current target velocity of the variable, $c_{i}$ are equilibrium points of linear acceleration fields with nonlinear basis functions $\psi _{i},\, g$ is the goal value (the target reach position), *K* and *D* are gain and damping parameters, and *u* is a phase variable which can be used to scale the duration of the movement. DMPs therefore generate a trajectory from $x_{0}$ to *g* that can be straight or parameterized to take any arbitrary path (which we do not exploit in this model but leave open the possibility for future work). In the reach module, the output of the DMP, $\hat{{x}}(t)$, is used as an actual target position for the wrist that is input to the inverse kinematics controller at each time step.

Given a desired wrist location, the inverse arm kinematics controller computes the required wrist displacement and then uses the pseudo-inverse of the Jacobian to compute the required joint rotations to bring the wrist to that position. The body’s Jacobian matrix describes how changes in shoulder $(\theta _{1},\, \theta _{2},\, \theta _{3})$ and elbow $(\theta _{4})$ angles result in changes in the wrist’s 3D position (*x*, *y*, *z*):

$$\begin{aligned} \left[ {{\begin{array}{l} {\dot{x}} \\ {\dot{y}} \\ {\dot{z}} \\ \end{array} }} \right] =\mathbf{J}\left[ {{\begin{array}{l} {\dot{\theta }_1 } \\ {\dot{\theta }_2 } \\ {\dot{\theta }_3 } \\ {\dot{\theta }_4 } \\ \end{array} }} \right] \end{aligned}$$

The inverse of the Jacobian matrix then describes how much each joint must rotate in order to effect a desired wrist displacement. The Jacobian is not invertible, so we use the pseudo-inverse:

$$\begin{aligned} \mathbf{J}^{+}=\mathbf{J}^\mathrm{T}\left( {\mathbf{JJ}^\mathrm{T}} \right) ^{-1} \end{aligned}$$

Each required joint rotation is used to input the target joint angle into the PD controller for that DOF:

$$\begin{aligned} \hat{{{\varvec{\theta }} }}=\varvec{\theta }+\mathbf{J}^{+}\left[ {{\begin{array}{l} {\dot{x}} \\ {\dot{y}} \\ {\dot{z}} \\ \end{array} }} \right] \end{aligned}$$

where $\dot{x}$, $\dot{y}$, $\dot{z}$ describe the desired wrist displacement.

The *grasp controller* controls the timing of the preshape and enclosure phases of the grasp. Depending on the phase of the grasp, the controller translates the selected virtual finger combination and maximum aperture into final target joint angles for each finger (see Online Resource 1, Table 2). Each virtual finger combination is associated with a preshape hand configuration with certain finger angles that can be modulated by the maximum aperture parameter, and a set of fingers to control during the enclose phase. The possible virtual finger combinations define the following grasps: precision pinch (index finger and thumb extended then enclosed), tripod grasp (index and middle fingers and thumb extended then enclosed), whole-hand prehension (all fingers and thumb extended then enclosed), and side grasps (all fingers enclosed and thumb extended then enclosed).

The grasp controller uses DMPs to control the timing of the preshape and enclosure phases of the grasp, but here the DMP output is interpreted as a normalized timing signal, rather than a physical target value. This is accomplished by setting the initial input to the DMP at 0 and the target to 1, and using its output at each time step to interpolate between the current finger joint angles and the final target angles in order to generate targets for the PD controllers. The preshape DMP is triggered as soon as a reach target is set, while the enclose DMP is triggered once the wrist reaches a certain threshold distance from the object, $\kappa$, or once the palm contacts the object, whichever happens first. ILGA thus captures a period of development where the innate infant grasp reflex is present, but some predictive hand enclosure during grasping starts to emerge.

### Parietal module: feature/affordance extraction

The populations in the Feature Extraction and Affordance Extraction modules are based on findings from a series of primate single-unit recording studies (Sakata et al. 1998; Galletti et al. 2003) in parietal cortex. These experiments found that neurons in the anterior intraparietal sulcus (AIP) are responsive to 3D features of objects relevant for manipulation (Murata et al. 2000). Neurons in the caudal intraparietal sulcus (cIPS) are selective for objects and their surfaces at preferred orientations. Subsets of these neurons have been described as axis orientation selective (AOS) and surface orientation selective (SOS, Taira et al. 1990). It has been suggested that the regions V6A and MIP are involved in encoding the direction of movement required to bring the arm to potential reach targets (Rizzolatti et al. 1998; Galletti et al. 2003). Both cIPS and V6A project to AIP (Shipp et al. 1998; Nakamura et al. 2001). Based on these studies, in ILGA the areas cIPS, MIP, and V6A extract object features and location and V6A/MIP and cIPS project this information to AIP for grasp affordance extraction.

**Fig. 6:**
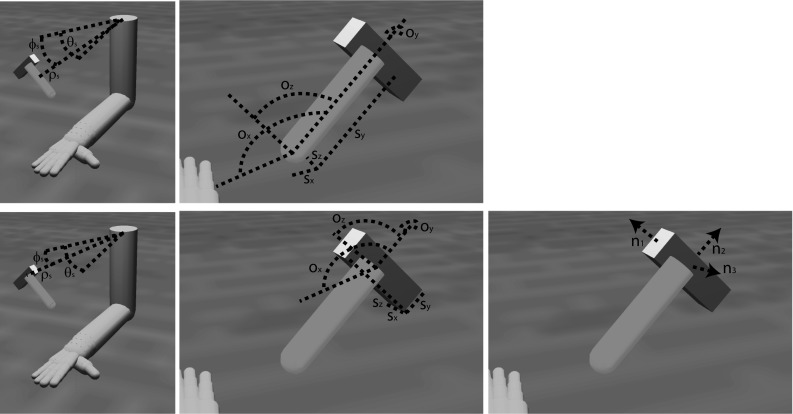
Object component variables represented in each parietal region for the handle (*top row*) and head (*bottom row*) of a hammer. The areas V6A and MIP represent the shoulder-centered direction of the center of the primitive, $\varphi _\mathrm{s}$ and $\theta _\mathrm{s}$, and the shoulder-centered distance, $\rho _\mathrm{s}$, and cIPS represents the object component’s orientation, $o_{x}$, $o_{y}$, $o_{z}$, size, $s_{x}$, $s_{y}$, $s_{z}$, and orientation of surface normal vectors (${n}_{1},\, {n}_{2}$, and ${n}_{3}$ in this case)

The neurophysiological experiments upon which the model parietal regions are based used simple objects (cube, cylinder, sphere, etc.) as stimuli. However, more complex objects such as hammers or coffee cups contain multiple affordances. We suggest that the dorsal visual stream analyzes objects in terms of a set of multiple affordances and accurately represents the metrics of different object components to provide data essential for grasp affordance extraction (Fig. 6).

#### V6A/MIP

The model region V6A/MIP represents the shoulder-centered direction and distance of each object component in spherical coordinates as two- and one-dimensional population codes, respectively. The module provides this information to parietal area AIP for affordance extraction and premotor area F2 for programming the reach. Area V6A contains mostly visual cells (Galletti et al. 1997; Rizzolatti et al. 1998) with receptive fields covering the whole visual field and representing each portion of it multiple times (Galletti et al. 1993, 1999). The so-called real-position cells are able to encode the spatial location of objects in the visual scene with visual receptive fields that remain anchored despite eye movements (Galletti et al. 1993). Intermingled with real-position cells are retinotopic cells, whose visual receptive fields shift with gaze, suggesting that the region is involved in converting coordinates from retinotopic to head- or body-centered reference frames (Galletti et al. 1993). Many neurons in V6A only respond when the arm is directed toward a particular region of space (Galletti et al. 1997; Fattori et al. 2005). Lesions of the region result in misreaching with the contralateral arm (Battaglini et al. 2002). It has thus been suggested that the region is involved in encoding the direction of movement in at least head-centered coordinates (but we use shoulder-centered coordinates for the single arm in the model) required to bring the arm to potential reach targets (Rizzolatti et al. 1998; Galletti et al. 2003). Area V6A receives input from central and peripheral visual field representations in V6 (Shipp et al. 1998) and projects to the premotor area F2 (Matelli et al. 1998; Shipp et al. 1998; Luppino et al. 2005).

Distance is represented in a one-dimensional population code, with each unit having a preferred distance, $\hat{{d}}_\mathrm{s}$ , uniformly distributed between 0 and 1 m. The activity of each unit, *i*, at time *t* is given by a Gaussian population code over the unit’s preferred distance and the actual shoulder-centered distance, $d_\mathrm{s}$, to each object component, *p*:

$$\begin{aligned} \mathbf{DIST}\left( {i,t} \right) =\sum _p {\left( {\text {e}^{-\frac{\left( {\hat{{d}}_\mathrm{s} \left( i \right) -d_\mathrm{s} \left( {p,t} \right) } \right) ^{2}}{2\sigma _\mathrm{DIST}^2 }}} \right) } +\varepsilon _\mathrm{DIST} \end{aligned}$$

where $\sigma _\mathrm{DIST}$ is the population code width and $\varepsilon _\mathrm{DIST}$ is a noise term. For all codes, we add a Gaussian noise term in order to simulate stochasticity in neural activity. A table of all parameter values is given in Online Resource 1, Table 3. Direction is encoded in a two-dimensional population code with each unit selective for both particular azimuth and elevation values of the shoulder-centered object direction:

$$\begin{aligned} \mathbf{DIR}\left( {i,j,t} \right) \!=\!\sum _p {\left( \! {\text {e}^{-\frac{\left( \! {\hat{{\theta }}_\mathrm{s} \left( {i,j} \right) -\theta _\mathrm{s} \left( {p,t} \right) } \right) ^{2}+\left( {\hat{{\varphi }}_\mathrm{s} \left( {i,j} \right) -\varphi _\mathrm{s} \left( {p,t} \right) } \right) ^{2}}{2\sigma _\mathrm{DIR}^2 }}} \right) } +\varepsilon _\mathrm{DIR} \end{aligned}$$

where $\theta _{s}(p,\, t)$ is the azimuth angle, ${\varphi }_{s}(p,\, t)$ is the elevation angle of object component *p* at time *t* in a shoulder-centered reference frame, $\sigma _\mathrm{DIR}$ is the population code width, and $\varepsilon _\mathrm{DIR}$ is a noise term. Each unit of the population, $i,\, j$, has preferred angles, $\hat{{\theta }}_\mathrm{s},\, \hat{{\varphi }}_\mathrm{s}$ , with $\hat{{\theta }}_\mathrm{s}$ uniformly distributed between 0 and $\pi$, and $\hat{{\varphi }}_\mathrm{s}$ uniformly distributed between $-\pi$ and 0.

#### cIPS

The model region cIPS contains three populations that represent the object orientation, size, and visible surface normal vectors as three-dimensional population codes. The caudal intraparietal sulcus (cIPS) is a region located the caudal part of the lateral bank and fundus of the intraparietal sulcus (Shikata et al. 1996). The cIPS receives input mainly from V3A, whose neurons are sensitive to binocular disparity and have small, retinotopic receptive fields (Sakata et al. 2005), and projects primarily to the anterior intraparietal sulcus (AIP, Nakamura et al. 2001). Neurons in area cIPS have large receptive fields (10–30 degrees in diameter) with no retinotopic organization (Tsutsui et al. 2005). Two functional classes of neurons in area cIPS have been described: surface-orientation-selective (SOS) neurons that are selective to the orientation of flat surfaces, and axis-orientation-selective (AOS) neurons that respond best to an elongated object whose principal axis is oriented in a particular direction. Both types of neurons respond best to binocular stimuli (Sakata et al. 1997) and are spatially intermingled (Nakamura et al. 2001). Muscimol-induced inactivation of this region disrupts performance on a delayed match-to-sample task with oriented surfaces using perspective and disparity cues (Tsutsui et al. 2001, 2005). Both types of cells include some neurons that are selective for the object’s dimensions (Kusunoki et al. 1993; Sakata et al. 1998). Again, these neurons have only been tested with simple objects. We suggest that they actually encode the features of object components that comprise complex objects. To simplify the model, we include AOS and SOS cells as well as one population that encodes the size of each object component.

*Axis-orientation-selective (AOS) cells* prefer bars tilted in the vertical, horizontal, or sagittal planes (Sakata et al. 1998, 1999). Some are selective for shape (rectangular versus cylindrical) and probably represent surface curvature (Sakata et al. 2005). Their discharge rate increases monotonically with object length, and their width response curve is monotonically decreasing in the 2–32 cm range. It is thought that these cells integrate orientation and width disparity cues to represent principal axis orientation (Sakata et al. 1998).

*Surface-orientation-selective (SOS) cells* are tuned to the surface orientation in depth of flat and broad objects (Shikata et al. 1996; Sakata et al. 1997, 1998, 1999). These cells respond to a combination of monocular and binocular depth cues (texture and disparity gradient cues) in representing surface orientation (Sakata et al. 2005). Neurons sensitive to multiple depth cues are widely distributed and spatially intermingled with those sensitive to only one depth cue (Tsutsui et al. 2005).

We model AOS cells as two subpopulations—one selective for rectangular and one for cylindrical objects. Each subpopulation is a three-dimensional population code, with each neuron *i*, *j*, *k*, selective for a combination of the components of the object’s main axis orientation, $o_{x}$, $o_{y}$, and $o_{z}$. When the object is cylindrical, the activity of each unit in the cylindrical AOS population is given by:

$$\begin{aligned}&\mathbf{CYL}\left( {i,j,k,t} \right) \\&\quad =\sum _p {\left( \text {e}^{-\frac{\left( {\hat{{o}}_x \left( {i,j,k} \right) -o_x \left( {p,t} \right) } \right) ^{2}+\left( {\hat{{o}}_y \left( {i,j,k} \right) -o_y \left( {p,t} \right) } \right) ^{2}+\left( {\hat{{o}}_z \left( {i,j,k} \right) -o_z \left( {p,t} \right) } \right) ^{2}}{2\sigma _\mathrm{CIPS}^2 }} \right) } \\&\qquad +\,\varepsilon _\mathrm{CIPS} \end{aligned}$$

where $\hat{{o}}_x \left( {i,j,k} \right)$, $\hat{{o}}_y \left( {i,j,k} \right)$, and $\hat{{o}}_z \left( {i,j,k} \right)$ are the preferred orientations of the unit *i*, *j*, *k* in the *x*, *y*, and *z* dimensions. When the object is not cylindrical, each unit’s activity in the cylindrical AOS population is given by the noise term. Given a rectangular object, the activity of each unit in the rectangular AOS population is defined by:

$$\begin{aligned}&\mathbf{RECT}\left( {i,j,k,t} \right) \\&\quad =\sum _p {\left( {\text {e}^{-\frac{\left( {\hat{{o}}_x \left( {i,j,k} \right) -o_x \left( {p,t} \right) } \right) ^{2}+\left( {\hat{{o}}_y \left( {i,j,k} \right) -o_y \left( {p,t} \right) } \right) ^{2}+\left( {\hat{{o}}_z \left( {i,j,k} \right) -o_z \left( {p,t} \right) } \right) ^{2}}{2\sigma _\mathrm{CIPS}^2 }}} \right) } \\&\qquad +\,\varepsilon _\mathrm{CIPS} \end{aligned}$$

Similarly, the noise term determines the activation of these units when the object is not rectangular.

We model the SOS population as a noisy three-dimensional Gaussian population code over the normal vector $(\mathbf{n}_{f},\, \mathbf{n}_{f}, \mathbf{n}_{f})$ of each visible surface, *f*, of a rectangular object component, *p*:

$$\begin{aligned}&\mathbf{SOS}\left( {i,j,k,t} \right) \\&\quad =\sum _p {\left( {\sum _f {\left( {\text {e}^{-\frac{\left( {\hat{{n}}_x \left( {i,j,k} \right) -\mathbf{n}_f \left( {p,x,t} \right) } \right) ^{2}+\left( {\hat{{n}}_y \left( {i,j,k} \right) -\mathbf{n}_f \left( {p,y,t} \right) } \right) ^{2}+\left( {\hat{{n}}_z \left( {i,j,k} \right) -\mathbf{n}_f \left( {p,z,t} \right) } \right) ^{2}}{2\sigma _\mathrm{CIPS}^2 }}} \right) } } \right) }\\&\qquad +\,\varepsilon _\mathrm{CIPS} \end{aligned}$$

Each unit has a preferred value in each dimension $(\hat{{n}}_x ,\,\hat{{n}}_y,\,\hat{{n}}_z )$ uniformly distributed between $-$1 and 1. If the object is not rectangular, the activity of each unit in these populations is determined by the noise term.

The size population, *S*, represents the size of an object component *p* in each dimension $(s_{x},\, s_{y},\, s_{z})$, with the activity of each unit, *i*, *j*, *k*, given by:

$$\begin{aligned}&\mathbf{S}\left( {i,j,k,t} \right) \\&\quad =\sum _p {\left( {\text {e}^{-\frac{\left( {\hat{{s}}_x \left( {i,j,k} \right) -s_x \left( {p,t} \right) } \right) ^{2}+\left( {\hat{{s}}_y \left( {i,j,k} \right) -s_y \left( {p,t} \right) } \right) ^{2}+\left( {\hat{{s}}_z \left( {i,j,k} \right) -s_z \left( {p,t} \right) } \right) ^{2}}{2\sigma _\mathrm{CIPS}^2 }}} \right) }\\&\qquad +\,\varepsilon _\mathrm{CIPS} \end{aligned}$$

where $\sigma _\mathrm{CIPS}$ is the population code width and $\varepsilon _\mathrm{CIPS}$ is a noise term.

#### AIP

The anterior intraparietal area AIP is located on the lateral bank of the anterior intraparietal sulcus and contains visually response neurons selective for 3D features of objects, motor-dominant neurons that only respond during grasping, and visuomotor neurons that are activated by grasping and modulated by sight of the object (Sakata et al. 1998). The region receives its main input from area cIPS (Nakamura et al. 2001), but also receives input from V6A (Shipp et al. 1998) and projects most strongly to the premotor region F5 (Borra et al. 2007).

In ILGA, the AIP module receives input from each population of the V6A/MIP and cIPS modules. While the distance to the object is important for parameterizing the reach as well as the coordination of the reach and grasp movements, it is not important for specifying the grasp itself, and therefore we do not model a connection between the distance population of V6A/MIP and AIP. The SOM is a toroidal grid of $40\times 40$ units, with the input vector, **I**, constructed by concatenating the activity vectors of each V6A/MIP and cIPS population into one vector, which is then normalized. The activity of each AIP unit with indices *i* and *j* is given by:

$$\begin{aligned} \mathbf{AIP}(i,j,t)=\mathbf{W}_\mathrm{AIP} (i,j,t)\mathbf{I}(t)+\varepsilon _\mathrm{AIP} \end{aligned}$$

The weights $\mathbf{W}_\mathrm{AIP}$ are initialized to small random values. Weight training uses a form of competitive learning. This learning algorithm modifies the connection weights of neurons in a neighborhood surrounding the neuron with the greatest activity level. The size of this neighborhood gets smaller over time and in ILGA, both the neighborhood size and learning rate are increased by the presence of the reinforcement signal. At the beginning of training, the neighborhood is broad and the learning rate is high. This causes the self-organization to take place on the global scale. As training progresses and the neighborhood size decreases, the weights converge to local estimate of the training input vectors. The modulation of the learning rate and neighborhood function by the global reinforcement signal ensures that input vectors that are used to plan stable grasps become represented by more units in the SOM at the expense of input vectors that result in failed grasps.

Given the input vector, **I**, the AIP unit with the most similar weight vector is determined as the best matching unit (BMU). The similarity metric we used was the Euclidean distance between the vectors. The weights of the BMU and all units within its “neighborhood” are adjusted in the direction of the input vector:

$$\begin{aligned} \mathbf{W}_\mathrm{AIP} \left( {i,j,t+1} \right)= & {} \mathbf{W}_\mathrm{AIP} \left( {i,j,t} \right) \\&+\,\Theta \left( {i,j,T} \right) \alpha \left( {t,T} \right) \left( \mathbf{I}( t)\right. \\&\left. -\,\mathbf{W}_\mathrm{AIP} \left( {i,j,t} \right) \right) \end{aligned}$$

where $\Theta$ is the reinforcement-dependent neighborhood function, T is the current training epoch, and $\alpha$ is the reinforcement-dependent learning rate. The neighborhood function is a Gaussian function over the Euclidean distance, $\beta$, between the indices of the neuron *i*, *j* and those of the BMU. The Gaussian is truncated at a certain radius, *r*, that also defines its spread:

$$\begin{aligned} \Theta \left( {i,j,t} \right) =\left\{ \begin{array}{ll} {\text {e}^{\frac{-\left( {\beta -0} \right) ^{2}}{2r( T )^{2}}}}&{} \quad {\text {if}}\,\,\beta <r( T ) \\ 0&{}\quad {\text {otherwise}} \\ \end{array} \right. \end{aligned}$$

The radius of the neighborhood function shrinks over the duration of training, but is expanded by the global reinforcement signal, *rs*(*t*):

$$\begin{aligned} r\left( {t,T} \right) =r_0 \text {e}^{\frac{-T}{\lambda }}+\text {rs}(t) \end{aligned}$$

where $r_{0}$ is the initial radius and the parameter $\lambda$ determines the rate at which the radius decreases (see Online Resource 1, Table 3 for parameter values). The learning rate also decreases over the duration of training:

$$\begin{aligned} \alpha \left( {t,T} \right) =\alpha _0 \text {e}^{\frac{-T}{\lambda }}+rs(t) \end{aligned}$$

where $\alpha _{0}$ is the initial learning rate.

### Premotor module: Reach and Grasp Planning

Each region in the Reach and Grasp Planning module contains one population of preparation-related cells and one population of execution-related cells. Execution-related cells discharge on movement onset, while signal-related cells show anticipatory activity prior to the start of movement. These broad categories of cells have been found in several premotor areas (Kurata 1994; Wise et al. 1997; Cisek and Kalaska 2002). In this model, preparation-related cells receive external input and project topologically to execution-related cells with hard-wired, fixed connections. Execution-related cells additionally receive tonic inhibition which is released when the go signal is detected, ensuring that the movement does not begin until the signal is observed. The basal ganglia are typically implicated in disinhibition of planned movements (Kropotov and Etlinger 1999) and reinforcement learning (Barto 1995). While models of the basal ganglia exist that could provide the tonic inhibition and reinforcement signals in ILGA (Gurney et al. 2001), we simply provide these inputs procedurally.

The Reach and Grasp Planning module contains several subpopulations based on various regions of dorsal and ventral premotor cortex including F2, F5, and F7. Each of these populations selects grasp motor parameters based on input from AIP and V6A/MIP. In ILGA, areas F2 and F7 are mainly involved with specifying the reach, with F2 selecting the center of the object component to reach to, and area F7 selecting an object-centered offset from that center for the reach target (Fig. 1). Area F5 selects the grasp type and maximal aperture, and F2/F5 selects the wrist orientation. All the motor parameters handled by the premotor cortex modules in both ILGM and ILGA are kinematics parameters—dynamics is completely ignored by these modules and handled entirely by the primary motor module.

Although there is evidence which supports the role of the primary motor cortex in both kinematic and dynamic encoding (Kalaska 2009), it has been shown that the evidence in favor of kinematic encoding could be an epiphenomenon of multidimensional muscle force coding (Todorov 2000). Lesions of the premotor cortex result in deficits in movement kinematics (Freund 1990; Gallese et al. 1994). However, neurons in dorsal and ventral premotor cortex have been found that correlate with movement dynamics variables (Xiao et al. 2006) and a series of studies has shown that the motor cortex may be involved in transforming kinematic variables from extrinsic to intrinsic reference frames (Kakei et al. 1999, 2001, 2003). Here, we make the simplifying assumption that the premotor cortex specifies movement kinematic variables which are translated into muscle forces by the primary motor cortex and spinal cord.

In this model, each preparation- and execution-related population is simulated as a DNF. Each DNF contained leaky integrator neurons with sigmoidal transfer functions. Each neuron consisted of a membrane potential variable, *u*, and a firing rate variable, *f*. The membrane potential was computed by integrating the weighted input over time with input from other neurons in the DNF:

$$\begin{aligned} \tau \frac{{\text {d}}\mathbf{u}}{{\text {d}}t}=-\mathbf{u}+h+\mathbf{IN}+\mathbf{f}*\mathbf{W}_\mathrm{DNF} +\varepsilon _\mathrm{DNF} \end{aligned}$$

where **u** and **f** are the population membrane potentials and firing rates, *h* is the baseline activation (see Online Resource 1, Table 4 for parameter values), **IN** is the weighted input, $*$ is the convolution operator, $\mathbf{W}_\mathrm{DNF}$ is the winner-take-all (WTA) weight kernel defined below, and $\varvec{\upvarepsilon }_\mathrm{DNF}$ is a noise term. The firing rate, *f*, is then a sigmoid function of the membrane potential:

$$\begin{aligned} \mathbf{f}=\frac{1}{1+\text {e}^{-\beta \left( {\mathbf{u}-u_0 } \right) }} \end{aligned}$$

where $\beta$ and $u_{0}$ are parameters of the sigmoid.

The weight kernel was set as a Gaussian that was negative except for a center peak, implementing the WTA functionality. The kernel was set to be twice as large as the population to ensure global competition. For one-dimensional DNFs, the weight kernel was given by:

$$\begin{aligned} \mathbf{W}_\mathrm{DNF} (i)=w_\mathrm{excite} \text {e}^{\frac{-\left( {i-N/2} \right) ^{2}}{2\sigma _\mathrm{DNF}^2 }}-w_\mathrm{inhibit} \end{aligned}$$

where $w_\mathrm{excite}$ is the height of the peak of the Gaussian, $w_\mathrm{inhibit}$ is the level of inhibition, *N* is the size of the population, and $\sigma _\mathrm{DNF}$ is the width of the Gaussian. Similarly, the two-dimensional weight kernel was defined as:

$$\begin{aligned} \mathbf{W}_\mathrm{DNF} \left( {i,j} \right) =w_\mathrm{excite} \text {e}^{\frac{-\left( {\left( {i-N/2} \right) ^{2}+\left( {j-N/2} \right) ^{2}} \right) }{2\sigma _\mathrm{DNF}^2 }}-w_\mathrm{inhibit} \end{aligned}$$

and the three-dimensional DNF was given by:

$$\begin{aligned} W_\mathrm{DNF} \left( {i,j,k} \right) \!=\!w_\mathrm{excite} \text {e}^{\frac{-\left( {\left( {i-N/2} \right) ^{2}\!+\!\left( {j-N/2} \right) ^{2}\!+\!\left( {k-N/2} \right) ^{2}} \right) }{2\sigma _\mathrm{DNF}^2 }}\!-\!w_\mathrm{inhibit} \end{aligned}$$

Every DNF in each modeled region used the same parameters (Table 3), which were determined empirically.

The tonic inhibitory input to each execution-related population, *GP*, was set to 10 before the go signal was detected and 0 once it appeared. Therefore, preparation-related cells in ILGA plan the movement, while the activation of execution-related cells triggers its onset. Reinforcement learning is applied to the afferent connection weights of the preparation-related cells, using the activity of the corresponding execution-related population as an eligibility trace. Eligibility traces are commonly used in reinforcement learning in order to assign credit to the appropriate connection weight for delayed reward (Singh and Sutton 1996). This is typically a decaying copy of the activated neurons, but since the delay between preparation-related cell activity and the achievement of a stable grasp that elicits a reward can be quite long, we use the activity of corresponding execution-related cells as the eligibility trace.

#### F2

Within the premotor cortex, the caudal portion F2 most likely codes reach movements in a shoulder-centered reference frame (Caminiti et al. 1991; Rizzolatti et al. 1998; Cisek and Kalaska 2002). Many of the cells in F2 have broad directional tuning, and their population activity appears to encode a vector representing the direction of arm movement and not the position of the end target (Weinrich and Wise 1982; Caminiti et al. 1991). The region was first defined by Matelli et al. (1985) and was later subdivided into the F2 dimple (F2d) and ventrorostral (F2vr) subregions (Matelli et al. 1998). Visual inputs to area F2 come mainly from the superior parietal lobe (Johnson et al. 1993; Caminiti et al. 1996). The subregion F2vr receives projections from area V6A (Shipp and Zeki 1995) and the medial intraparietal area MIP (Matelli et al. 1998; Shipp et al. 1998; Marconi et al. 2001). The main output of F2 projects to F1 (Dum and Strick 2005).

Area F2 contains a rostro-caudal gradient of cell types with preparation-related cells found predominantly in F2vr and execution-related cells located in F2d, the caudal portion adjacent to F1 (Tanne et al. 1995; Johnson et al. 1996). Preparation-related cells are 43 % of F2 neurons and respond to the visual target for reaching, while execution-related cells have changes in activity that are synchronized with the onset of movement (Weinrich and Wise 1982). Some execution-related cells are only active after the go signal and these are more common caudally (Crammond and Kalaska 2000). This categorization of cells seems to correspond to a similar modality-based classification used by Fogassi et al. (1999) and Raos et al. (2004), which describes cells as purely motor, visually modulated, or visuomotor. Purely, motor cells are not affected by object presentation or visual feedback of the hand, visually modulated cells discharge differentially when reaching in the light vs. dark, and visuomotor cells discharge during object fixation without movement. Most of visually modulated or visuomotor cells are in F2vr (Fogassi et al. 1999) and therefore likely correspond to the preparation-related cells described by Crammond and Kalaska (2000). Our model thus subdivides F2 into rostral and caudal regions (F2vr and F2d, respectively) and simplifies the distribution of cell types by confining preparation-related cells to the rostral region and execution-related cells to the caudal region.

Most cells in F2 are sensitive to amplitude and direction, with very few cells sensitive to only amplitude (Fu et al. 1993; Messier and Kalaska 2000). However, muscimol inactivation caused increases in directional errors when conditional cues are presented, but amplitude and velocity were unchanged (Kurata and Hoffman 1994). Neurons in the dorsal premotor cortex have more recently been shown to encode the relative position of the eye, hand, and goal (Pesaran et al. 2006), but we do not vary the eye position in these simulations and this influence is therefore constant. We thus decode the output of F2d as a population code with each cell having a preferred spherical coordinate in a shoulder-centered reference frame. Note that the issue of which reference frame is used in the reach circuit is still debated.

We model the F2vr region as two DNFs encoding the shoulder-centered direction (**F2vrDIR**) and distance (**F2vrRAD**) of the target object in spherical coordinates. The input to each DNF is given by:

$$\begin{aligned} \mathbf{IN}_\mathrm{F2vrDIR} (t)= & {} \mathbf{DIR}(t)\mathbf{W}_{\mathrm{DIR}\rightarrow \mathrm{F2}} +\varvec{\varepsilon }_\mathrm{F2}\\ \mathbf{IN}_\mathrm{F2vrRAD} (t)= & {} \mathbf{DIST}(t)\mathbf{W}_{\mathrm{DIST}\rightarrow \mathrm{F2}} +\varvec{\varepsilon }_\mathrm{F2} \end{aligned}$$

where the matrices $\mathbf{W}_{\mathrm{DIR}\rightarrow \mathrm{F2}}$ and $\mathbf{W}_{\mathrm{DIST}\rightarrow \mathrm{F2}}$ define the weights of the projections from V6A/MIP to F2. Since we assume that reaching ability has already developed, these weights are not subject to learning and set according to the following rule:

$$\begin{aligned} \mathbf{W}\left( {i,j} \right) =3\mathbf{I} \end{aligned}$$

where **I** is the identity matrix. This results in F2vr faithfully selecting the center of the object component (as signaled by V6A/MIP) as the position from which to calculate the final target for the wrist using the object-centered reach offset. The F2d region is similarly modeled as two DNFs that each receive excitatory input from F2vr and tonic inhibitory input, *GP*:

$$\begin{aligned}&\mathbf{IN}_\mathrm{F2dDIR} (t)=\mathbf{F2vrDIR}(t)\mathbf{W}_{\mathrm{F2}\rightarrow \mathrm{F2}} +\mathbf{GP}(t)+\varvec{\varepsilon }_{\mathrm{F2}}\\&\mathbf{IN}_\mathrm{F2dRAD} (t)=\mathbf{F2vrRAD}(t)\mathbf{W}_{\mathrm{F2}\rightarrow \mathrm{F2}} +\mathbf{GP}(t)+\varvec{\varepsilon }_{\mathrm{F2}} \end{aligned}$$

The weight matrices between preparation- and execution-related premotor populations, $\mathbf{W}_{\mathrm{F2}\rightarrow \mathrm{F2}}$, were not subject to learning and were set as follows:

$$\begin{aligned} \mathbf{W}\left( {i,j} \right) =2\mathbf{I} \end{aligned}$$

#### F7

While there does not appear to be direct evidence for a population of premotor neurons encoding an object-centered reach offset, there is some suggestion that such a representation does exist and may be located in the dorsal premotor cortex. The rostral portion of the dorsal premotor cortex, area F7 (approximately equal to PMdr, Wise et al. 1997), can be separated into the dorso-rostral supplementary eye field (SEF) and a lesser-known ventral region. The SEF is known to contain neurons which encode space in an object-centered reference frame (Olson and Gettner 1995), but the region is implicated in control of eye movements. While the properties of ventral F7 are not well known, it does contain neurons related to arm movements (Fujii et al. 1996, 2002), receives the same thalamic input as the arm region of F6, and receives input from the same region of the superior temporal sulcus that projects to F2vr (Rizzolatti and Luppino 2001). The ventral portion of F7 may therefore be a likely candidate for the location of population of neurons encoding reach targets in an object-centered frame of reference.

We model F7 as a preparation- and execution-related population. The preparation-related population consists of two DNFs encoding the object-centered reach offset in spherical coordinates (**F7sDIR** encoding azimuth and elevation, and **F7sRAD** encoding the radius). Inputs to the F7 module come from F2, signaling the center of the object component, and AIP, providing an affordance representation. The input to each preparation-related DNF is given by:

$$\begin{aligned} \mathbf{IN}_\mathrm{F7sDIR} (t)= & {} \mathbf{AIP}(t)\mathbf{W}_{\mathrm{AIP}\rightarrow \mathrm{F7DIR}} (t)\\&+\,\mathbf{F2vrDIR}(t)\mathbf{W}_{\mathrm{F2}\rightarrow \mathrm{F7}} +\varvec{\varepsilon }_\mathrm{F7} \end{aligned}$$

$$\begin{aligned} \mathbf{IN}_\mathrm{F7sRAD} (t)= & {} \mathbf{AIP}(t)\mathbf{W}_{\mathrm{AIP}\rightarrow \mathrm{F7RAD}} (t)+\varvec{\varepsilon }_\mathrm{F7} \end{aligned}$$

The execution-related population also contains two DNFs, each corresponding to one DNF in the preparation-related population. The input to each execution-related DNF is given by:

$$\begin{aligned}&\mathbf{IN}_{\mathrm{F7eDIR}} (t)=\mathbf{F7sDIR}(t)\mathbf{W}_{\mathrm{F7}\rightarrow \mathrm{F7}} (t)+\mathbf{GP}(t)+\varvec{\varepsilon }_{\mathrm{F7}}\\&\mathbf{IN}_\mathrm{F7eRAD} (t)=\mathbf{F7sRAD}(t)\mathbf{W}_{\mathrm{F7}\rightarrow \mathrm{F7}} (t)+\mathbf{GP}(t)+\varvec{\varepsilon }_{\mathrm{F7}} \end{aligned}$$

The connection weights between F2 and AIP and the preparation-related F7 populations were initialized to small random values and subject to learning using a variant of the REINFORCE rule (Sutton and Barto 1998) which is Hebbian for positive reward values and anti-Hebbian for negative ones:

$$\begin{aligned}&\mathbf{W}_{\mathrm{AIP}\rightarrow \mathrm{F7DIR}} \left( {a,b,i,j,t+1} \right) =\mathbf{W}_{\mathrm{AIP}\rightarrow \mathrm{F7DIR}} \left( {a,b,i,j,t} \right) \\&\quad +\,\alpha _\mathrm{F7} rs(t)\left( {\mathbf{AIP}\left( {a,b,t} \right) \mathbf{F7eDIR}\left( {i,j,t} \right) } \right) \\&\mathbf{W}_{\mathrm{AIP}\rightarrow \mathrm{F7RAD}} \left( {a,b,i,t+1} \right) =\mathbf{W}_{\mathrm{AIP}\rightarrow \mathrm{F7RAD}} \left( {a,b,i,t} \right) \\&\quad +\,\alpha _\mathrm{F7} rs(t)\left( {\mathbf{AIP}\left( {a,b,t} \right) \mathbf{F7eRAD}\left( {i,t} \right) } \right) \\&\mathbf{W}_{\mathrm{F2}\rightarrow \mathrm{F7}} \left( {a,b,i,j,t+1} \right) =\mathbf{W}_{\mathrm{F2}\rightarrow \mathrm{F7}} \left( {a,b,i,j,t} \right) \\&\quad +\,\alpha _{\mathrm{F7}} rs(t)\left( {\mathbf{F2dDIR}\left( {a,b,t} \right) \mathbf{F7eDIR}\left( {i,j,t} \right) } \right) \end{aligned}$$

The outputs of the execution-related populations are used as the eligibility traces since in general the object may not be visible at the end of the grasp and preparation-related cells may not be active anymore.

#### F5

Many neurons in premotor area F5 fire in association with specific types of manual action, such as precision grip, finger prehension, and whole-hand prehension (Rizzolatti and Camarda 1988) as well as tearing and holding. Some neurons in F5 discharge only during the last part of grasping; others start to fire during the phase in which the hand opens and continue to discharge during the phase when the hand closes; finally a few discharge prevalently in the phase in which the hand opens. Grasping appears therefore to be coded by the joint activity of populations of neurons, each controlling different phases of the motor act. Raos et al. (2006) found that F5 neurons selective for both grip type and wrist orientation maintained this selectivity when grasping in the dark. Simultaneous recording from F5 and F1 showed that F5 neurons were selective for grasp type and phase, while an F1 neuron might be active for different phases of different grasps (Umilta et al. 2007). This suggests that F5 neurons encode a high-level representation of the grasp motor schema, while F1 neurons (or, at least, some of them) encode the component movements or components of a population code for muscle activity of each grasp phase.

We model F5 as a preparation- and execution-related population, each containing a one-dimensional DNF for each VF combination with neurons in each DNF selective for maximum grasp aperture. In this module, in addition to the WTA dynamic within DNFs, every unit in a DNF laterally inhibits every other unit in the other DNFs, so that inter-DNF competition selects a VF combination, while intra-DNF competition selects a maximum aperture. The possible VF combinations are index finger pad–thumb pad (precision grasp), index+middle finger pads–thumb pad (tripod grasp), inner fingers–palm (power grasp), and thumb pad–side of index finger (side grasp). The maximal aperture is encoded as a normalized value from 0 to 1 that is transformed into target finger joint angles by the grasp motor controller (see Online Resource 1, Table 2).

Inputs to the F5 module come from the AIP module, and therefore F5 selects an appropriate grasp based on the learned affordance representation. The inputs to each preparation-related population, **F5sPREC**, **F5sTRI**, **F5sPOW**, **F5sSIDE** for the precision, tripod, power, and side grasps, respectively, are given by:

$$\begin{aligned} \mathbf{IN}_\mathrm{F5sPREC} (t)= & {} \mathbf{AIP}(t)\mathbf{W}_{\mathrm{AIP}\rightarrow \mathrm{F5PREC}} (t)\\&-\,\mathbf{W}_{\mathrm{F5s}\rightarrow \mathrm{F5s}} \sum _i \left( \mathbf{F5sTRI}\left( {i,t} \right) \right. \\&\left. +\,\mathbf{F5sPOW}\left( {i,t} \right) +\mathbf{F5sSIDE}\left( {i,t} \right) \right) +\varvec{\varepsilon }_\mathrm{F5} \end{aligned}$$

$$\begin{aligned} \mathbf{IN}_\mathrm{F5sTRI} (t)= & {} \mathbf{AIP}(t)\mathbf{W}_{\mathrm{AIP}\rightarrow \mathrm{F5TRI}} (t)\\&-\,\mathbf{W}_{\mathrm{F5s}\rightarrow \mathrm{F5s}} \sum _i \left( \mathbf{F5sPREC}\left( {i,t} \right) \right. \\&\left. +\,\mathbf{F5sPOW}\left( {i,t} \right) +\mathbf{F5sSIDE}\left( {i,t} \right) \right) +\varvec{\varepsilon }_\mathrm{F5} \\ \mathbf{IN}_\mathrm{F5sPOW} (t)= & {} \mathbf{AIP}(t)\mathbf{W}_{\mathrm{AIP}\rightarrow \mathrm{F5POW}} (t)\\&-\,\mathbf{W}_{\mathrm{F5s}\rightarrow \mathrm{F5s}} \sum _i \left( \mathbf{F5sPREC}\left( {i,t} \right) \right. \end{aligned}$$

$$\begin{aligned}&+\,\left. \mathbf{F5sTRI}\left( {i,t} \right) +\mathbf{F5sSIDE}\left( {i,t} \right) \right) +\varvec{\varepsilon }_{\mathrm{F5}} \\ \mathbf{IN}_{\mathrm{F5sSIDE}} (t)= & {} \mathbf{AIP}(t)\mathbf{W}_{\mathrm{AIP}\rightarrow \mathrm{F5SIDE}} (t)\\&-\,\mathbf{W}_{\mathrm{F5s}\rightarrow \mathrm{F5s}} \sum _i \left( \mathbf{F5sPREC}\left( {i,t} \right) \right. \\&\left. +\,\mathbf{F5sTRI}\left( {i,t} \right) +\mathbf{F5sPOW}\left( {i,t} \right) \right) +\varvec{\varepsilon }_{\mathrm{F5}} \end{aligned}$$

where $\mathbf{W}_{\mathrm{F5s}\rightarrow \mathrm{F5s}}$ is the inhibitory connection weight between DNFs, set to .25 in these simulations. The inputs to the execution-related populations, **F5ePREC**, **F5eTRI**, **F5ePOW**, **F5eSIDE**, are given by:

$$\begin{aligned}&\mathbf{IN}_\mathrm{F5ePREC} (t)=\mathbf{F5sPREC}(t)\mathbf{W}_{\mathrm{F5s}\rightarrow \mathrm{F5e}} (t)+\mathbf{GP}(t)+\varvec{\varepsilon }_\mathrm{F5}\\&\mathbf{IN}_\mathrm{F5eTRI} (t)=\mathbf{F5sTRI}(t)\mathbf{W}_{\mathrm{F5s}\rightarrow \mathrm{F5e}} (t)+\mathbf{GP}(t)+\varvec{\varepsilon }_\mathrm{F5}\\&\mathbf{IN}_\mathrm{F5ePOW} (t)=\mathbf{F5sPOW}(t)\mathbf{W}_{\mathrm{F5s}\rightarrow \mathrm{F5e}} (t)+\mathbf{GP}(t)+\varvec{\varepsilon }_\mathrm{F5}\\&\mathbf{IN}_\mathrm{F5eSIDE} (t)=\mathbf{F5sSIDE}(t)\mathbf{W}_{\mathrm{F5s}\rightarrow \mathrm{F5e}} (t)+\mathbf{GP}(t)+\varvec{\varepsilon }_\mathrm{F5} \end{aligned}$$

The $\mathbf{W}_{\mathrm{F5s}\rightarrow \mathrm{F5e}}$ weights were set just as the $\mathbf{W}_{\mathrm{F2}\rightarrow \mathrm{F2}}$ and $\mathbf{W}_{\mathrm{F7}\rightarrow \mathrm{F7}}$ weights, and the connection weights $\mathbf{W}_{\mathrm{AIP}\rightarrow \mathrm{F5PREC}}$, $\mathbf{W}_{\mathrm{AIP}\rightarrow \mathrm{F5TRI}}$, $\mathbf{W}_{\mathrm{AIP}\rightarrow \mathrm{F5POW}}$, and $\mathbf{W}_{\mathrm{AIP}\rightarrow \mathrm{F5SIDE}}$ were initialized to small random values and subject to learning using the REINFORCE learning rule (as the connections to F7, Sutton and Barto 1998):

$$\begin{aligned}&\mathbf{W}_{\mathrm{AIP}\rightarrow \mathrm{F5PREC}} \left( {a,b,i,t+1} \right) =\mathbf{W}_{\mathrm{AIP}\rightarrow \mathrm{F5PREC}} \left( {a,b,i,t} \right) \\&\quad +\,\alpha _\mathrm{F5} rs(t)\left( {\mathbf{AIP}\left( {a,b,t} \right) \mathbf{F5ePREC}\left( {i,t} \right) } \right) \\&\mathbf{W}_{\mathrm{AIP}\rightarrow \mathrm{F5TRI}} \left( {a,b,i,t+1} \right) =\mathbf{W}_{\mathrm{AIP}\rightarrow \mathrm{F5TRI}} \left( {a,b,i,t} \right) \\&\quad +\,\alpha _\mathrm{F5} rs(t)\left( {\mathbf{AIP}\left( {a,b,t} \right) \mathbf{F5eTRI}\left( {i,t} \right) } \right) \\&\mathbf{W}_{\mathrm{AIP}\rightarrow \mathrm{F5POW}} \left( {a,b,i,t+1} \right) =\mathbf{W}_{\mathrm{AIP}\rightarrow \mathrm{F5POW}} \left( {a,b,i,t} \right) \\&\quad +\,\alpha _\mathrm{F5} rs(t)\left( {\mathbf{AIP}\left( {a,b,t} \right) \mathbf{F5ePOW}\left( {i,t} \right) } \right) \\&\mathbf{W}_{\mathrm{AIP}\rightarrow \mathrm{F5SIDE}} \left( {a,b,i,t+1} \right) =\mathbf{W}_{\mathrm{AIP}\rightarrow \mathrm{F5SIDE}} \left( {a,b,i,t} \right) \\&\quad +\,\alpha _\mathrm{F5} rs(t)\left( {\mathbf{AIP}\left( {a,b,t} \right) \mathbf{F5eSIDE}\left( {i,t} \right) } \right) \end{aligned}$$

#### Wrist rotation

Infants starting at 7 months old begin to pre-orient their hands to match an object’s affordances when reaching for that object (Witherington 2005). By 9 months old, infants are skilled at hand pre-orientation and adjustment and increase reach and grasp efficiency (Morrongiello and Rocca 1989). Neurons have been described in area F2 that become active in relation to specific orientations of visual stimuli and to corresponding hand/wrist movements (Raos et al. 2004). That same paper showed that 66 % of grasp neurons in F2 were highly selective for grasp type and that 72 % were highly selective for wrist orientation. In addition to reach target selection, the dorsal premotor cortex is implicated in wrist movements (Riehle and Requin 1989; Kurata 1993). Raos et al. (2006) show that F5 neurons combine selectivity for grip type and wrist orientation, and that 21 out of the 38 they tested for wrist orientation selectivity showed high selectivity for a particular orientation. The most plausible hypothesis that reconciles these findings is that the dorsal premotor cortex is involved in coding reach direction and the ventral premotor cortex is involved in coding grasps, and that interconnections between F2 and F5 (Marconi et al. 2001) allow the two regions to converge on a wrist orientation appropriate for the selected reach direction and grasp type.

We model the F2/F5 wrist rotation network as a preparation- and an execution-related population, similarly to the other premotor modules. The signal-related population contains a three-dimensional DNF, with each unit selective for a combination of the angles of the DOFs of the wrist within its joint angle limits (see Online Resource 1, Table 1). The F2/F5 module receives inputs from the AIP, F5, and F7 module and therefore selects the wrist orientation based on the affordance representation and selected grasp and reach offset. The input to the three-dimensional preparation-related DNF is given by:

$$\begin{aligned}&\mathbf{IN}_\mathrm{WRs} (t)=\mathbf{AIP}(t)\mathbf{W}_{\mathrm{AIP}\rightarrow \mathrm{WR}} (t)+\mathbf{F7sDIR}(t)\\&\quad \times \mathbf{W}_{\mathrm{F7}\rightarrow \mathrm{WR}} (t)+\,\mathbf{F5sPREC}(t)\mathbf{W}_{\mathrm{F5PREC}\rightarrow \mathrm{WR}} (t) \\&\quad +\mathbf{F5sTRI}(t)\mathbf{W}_{\mathrm{F5TRI}\rightarrow \mathrm{WR}} (t)+\,\mathbf{F5sPOW}(t)\\&\quad \times \mathbf{W}_{\mathrm{F5POW}\rightarrow \mathrm{WR}} (t)+\mathbf{F5sSIDE}(t)\mathbf{W}_{\mathrm{F5SIDE}\rightarrow \mathrm{WR}} (t)\\&\quad + \varvec{\varepsilon }_{\mathrm{WR}} \end{aligned}$$

Note that the preparation-related F2/F5 DNF receives input from the *F7sDIR* population encoding the direction of the reach offset, but it does not get reach offset radius information from the *F7sRAD* population since the wrist rotation should not depend on the offset radius. The execution-related population also contains a three-dimensional DNF, with its input given by:

$$\begin{aligned} \mathbf{IN}_\mathrm{WRe} (t)=\mathbf{WRs}(t)\mathbf{W}_{\mathrm{WR}\rightarrow \mathrm{WR}} +\mathbf{GP}(t)+\varvec{\varepsilon }_{\mathrm{WR}} \end{aligned}$$

As with F5 and F7, the weights of the afferent connections of the preparation-related populations were updated using the REINFORCE learning rule (Sutton and Barto 1998):

$$\begin{aligned}&\mathbf{W}_{\mathrm{AIP}\rightarrow \mathrm{WR}} \left( {a,b,i,j,k,t+1} \right) =\mathbf{W}_{\mathrm{AIP}\rightarrow \mathrm{WR}} \left( {a,b,i,j,k,t} \right) \\&\quad +\,\alpha _{\mathrm{WR}} rs(t)\left( {\mathbf{AIP}\left( {a,b,t} \right) \mathbf{WRe}\left( {i,j,k,t} \right) } \right) \\&\mathbf{W}_{\mathrm{F7}\rightarrow \mathrm{WR}} \left( {a,b,i,j,k,t+1} \right) =\mathbf{W}_{\mathrm{AIP}\rightarrow \mathrm{F7}} \left( {a,b,i,j,k,t} \right) \\&\quad +\,\alpha _{\mathrm{WR}} rs(t)\left( {\mathbf{F7eDIR}\left( {a,b,t} \right) \mathbf{WRe}\left( {i,j,k,t} \right) } \right) \\&\mathbf{W}_{\mathrm{F5PREC}\rightarrow \mathrm{WR}} \left( {a,i,j,k,t+1} \right) \!\!=\!\! \mathbf{W}_{\mathrm{F5PREC}\rightarrow \mathrm{WR}} \left( {a,i,j,k,t} \right) \\&\quad +\,\alpha _{\mathrm{WR}} rs(t)\left( {\mathbf{F5ePREC}\left( {a,t} \right) \mathbf{WRe}\left( {i,j,k,t} \right) } \right) \\&\mathbf{W}_{\mathrm{F5TRI}\rightarrow \mathrm{WR}} \left( {a,i,j,k,t+1} \right) =\mathbf{W}_{\mathrm{F5TRI}\rightarrow \mathrm{WR}} \left( {a,i,j,k,t} \right) \\&\quad +\,\alpha _{\mathrm{WR}} rs(t)\left( {\mathbf{F5eTRI}\left( {a,t} \right) \mathbf{WRe}\left( {i,j,k,t} \right) } \right) \\&\mathbf{W}_{\mathrm{F5POW}\rightarrow \mathrm{WR}} \left( {a,i,j,k,t+1} \right) \!\!=\!\!\mathbf{W}_{\mathrm{F5POW}\rightarrow \mathrm{WR}} \left( {a,i,j,k,t} \right) \\&\quad +\,\alpha _{\mathrm{WR}} rs(t)\left( {\mathbf{F5ePOW}\left( {a,t} \right) \mathbf{WRe}\left( {i,j,k,t} \right) } \right) \\&\mathbf{W}_{\mathrm{F5SIDE}\rightarrow \mathrm{WR}} \left( {a,i,j,k,t+1} \right) \!\!=\!\!\mathbf{W}_{\mathrm{F5SIDE}\rightarrow \mathrm{WR}} \left( {a,i,j,k,t} \right) \\&\quad +\,\alpha _{\mathrm{WR}} rs(t)\left( {\mathbf{F5eSIDE}\left( {a,t} \right) \mathbf{WRe}\left( {i,j,k,t} \right) } \right) \end{aligned}$$

### Training

Each training trial was run for 5 s with a 1-ms time step. All training simulations used the same protocol in which at 0.5 s a green object appeared in the model’s field of view. Different objects were presented (cubes, rectangular prisms, cylinders, spheres, and flat plates) at random orientations and locations. The model input was obtained by getting the object’s shape, color, size, orientation, and position from the physics simulator. At 1 s into the simulation the object turned red, triggering the release of inhibition from the execution-related premotor populations by setting the tonic inhibitory input *GP* to each of these populations to 0. In the last five time steps of each trial (corresponding to 5 ms of simulation time), *rs*(*t*) is set to ${\text {DA}}_\mathrm{success}$ if the grasp is successful, and ${\text {DA}}_\mathrm{fail}$ if not. Although the bulk of learning in this model is done simultaneously in all layers, we still found it necessary to use a somewhat staged learning approach to bootstrap the system. During AIP pretraining trials, no movements were attempted and *rs*(*t*) was always equal to 0. During the wrist rotation pretraining trials, *rs*(*t*) was set to $\frac{{\text {DA}}_\mathrm{success} }{4}$ if palm contact was achieved at all.

#### AIP pretraining

We found that at the start of training, the neurons in AIP did not have sufficient activity rates to drive the premotor populations. This resulted in approximately 1000 trials in which no grasps were attempted, but the connection weights into AIP were slowly modified according to the SOM weight adjustment rule described above. Eventually large numbers of AIP units were significantly activated in widespread overlapping representations sufficient to activate premotor populations. This period may correspond to a period of visual experience before the development of skilled reaching in which no grasps are attempted and visual regions are shaped by unsupervised learning mechanisms.

Although we assume the existence of a pretrained reaching circuit, this period could correspond to a period of motor babbling in which internal models of the arm are learned (Bullock et al. 1993). This period of training may also correspond to the motor babbling period of infant development modeled by Kuperstein (1988).

#### Wrist rotation pretraining

**Fig. 7:**
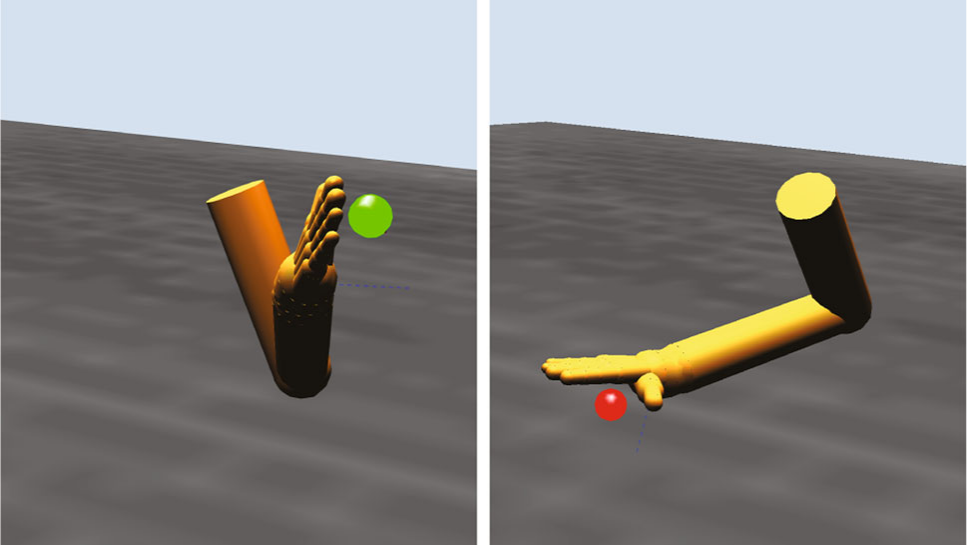
Examples of two successful reaches in the wrist rotation pretraining stage. Note the rotation of the wrist which orients the palm toward the object

The added realism of our simulator compared to that of ILGM comes at the price of a much lower probability of successfully grasping an object with random motor parameters. This makes the task of learning much more difficult. To surmount this problem, we used a period of pretraining in which any palm contact was rewarded and only the connection weights between F7 and F2/F5 were modified. At the end of pretraining, the system could at least orient the hand in the correct direction to make finger or palm contact with the object at various locations and orientations (Fig. 7), similar to the automatic wrist orienting mechanism used in some ILGM simulations. This period of training therefore corresponds to infant development from 7 to 9 months where infants learn to pre-orient their wrist in response to an object’s affordances (Morrongiello and Rocca 1989; Witherington 2005).

#### Grasp training

After pretraining, the system was trained for 10,000 trials with different objects at random locations and orientations. The object type, position, and orientation were changed every 6 trials since each trial lasted 5 s and infants will repeatedly reach to a novel object for at least 30 s before habituation (Von Hofsten and Spelke 1985). During these training trials, only stable grasps were positively reinforced and all modifiable connection weights (dashed arrows in Fig. 4) were subject to learning.

## Results

After training, the model was able to generate stable grasps of each object type at various locations and orientations. The representation in AIP allowed the model to generalize across object properties enough to successfully grasp objects in novel configurations in a few attempts. Here, we demonstrate the ability of the model to generate successful grasps, analyze the learned representations in AIP, and generate predictions for future experiments.

### AIP representation

**Fig. 8:**
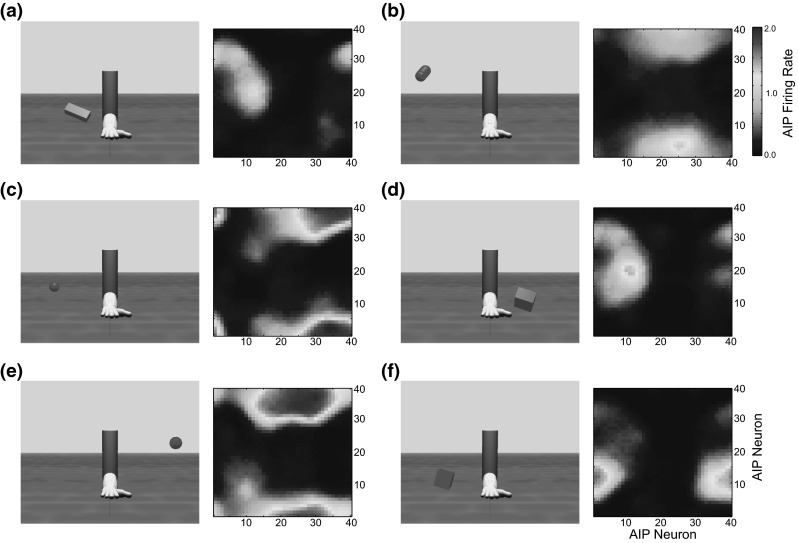
AIP activation after pretraining for different objects in various locations and orientations. *Each panel* shows a third-person view (*left*) and AIP activation (*right*)

Each object at each location and orientation in the training set elicited a slightly different activation pattern in the cIPS and V6A/MIP populations. However after pretraining AIP, objects with similar features elicited similar, overlapping, patterns of activation in this region (Fig. 8). This is an inherent property of SOMs and is what allows the model to successfully grasp novel objects in familiar locations and orientations (see Grasp training, below).

Murata et al. (2000) tested the response of AIP neurons to the sight of various types of objects and found that a high proportion of visually responsive AIP neurons were highly shape selective, responding strongly to one particular object shape and weakly to all others. They used multidimensional scaling (MDS) to look at how moderately object-selective neurons encode the similarity of objects. MDS is a method of reducing a high-dimensional input space into a lower-dimensional space while preserving topological relations between vectors. It was found that moderately object-selective visually responsive AIP neurons respond to common combinations of geometric features shared by similar objects such as shape, size, and/or orientation. However, note that the objects used in this experiment were not natural, complex objects, but geometric primitives. We suggest that AIP codes combinations of features of object components and that given a complex object, AIP neurons selective for features of each of its components will be activated.

**Fig. 9:**
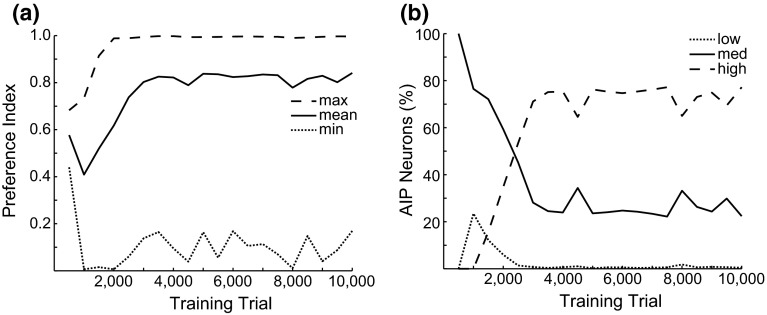
**a** Object shape specificity statistics for the AIP population during training (*solid* maximum PI, *dashed* mean PI, *dotted* minimum PI). **b** Numbers of highly (*solid*), moderately (*dashed*), and non- (*dotted*) shape-specific neurons throughout training

We did not include any explicit encoding of object shape in the inputs to AIP (although the CYL and RECT populations selectively respond to features of particularly shaped objects). However, we found that after training AIP contained a mixture of highly, moderately, and weakly shape-selective neurons (Fig. 9). To characterize a neuron’s object preference, we used the same technique as Raos et al. (2006) where the object specificity of a neuron is defined as a preference index (PI) :

$$\begin{aligned} \text {PI}=\frac{n-\left( {\frac{\sum {r_i } }{r_\mathrm{pref} }} \right) }{n-1} \end{aligned}$$

where *n* is the number of object shapes tested, $r_{i}$ is the mean activity of the neuron for objects with shape *i*, and $r_\mathrm{pref}$ is the mean activity for the preferred object shape in the current epoch. This measure can range from 0 to 1, with 0 meaning the neuron responds equally to all object shapes and 1 indicating activity for only one shape. We classified neurons as highly shape selective if they had a PI greater than .75, moderately selective if they had a PI between .25 and .75, and non-shape selective if they had a PI less than .25. The PI of each neuron was evaluated in blocks of 500 trials throughout the entire training period.

We found that at the start of training, all AIP neurons were moderately shape selective (Fig. 9b), responding to combinations of features such as size and orientation. After about 500 trials, a small amount of neurons became non-shape selective and simply responded to the presence of any object shape. By the end of the AIP pretraining period, nearly 80 % of AIP neurons were highly shape selective, some reaching a maximum PI near 1.0, indicating they only responded to specific combinations of features that signaled a particular object shape. This selectivity was maintained throughout the entire training period, even after the model began to attempt grasps.

**Fig. 10:**
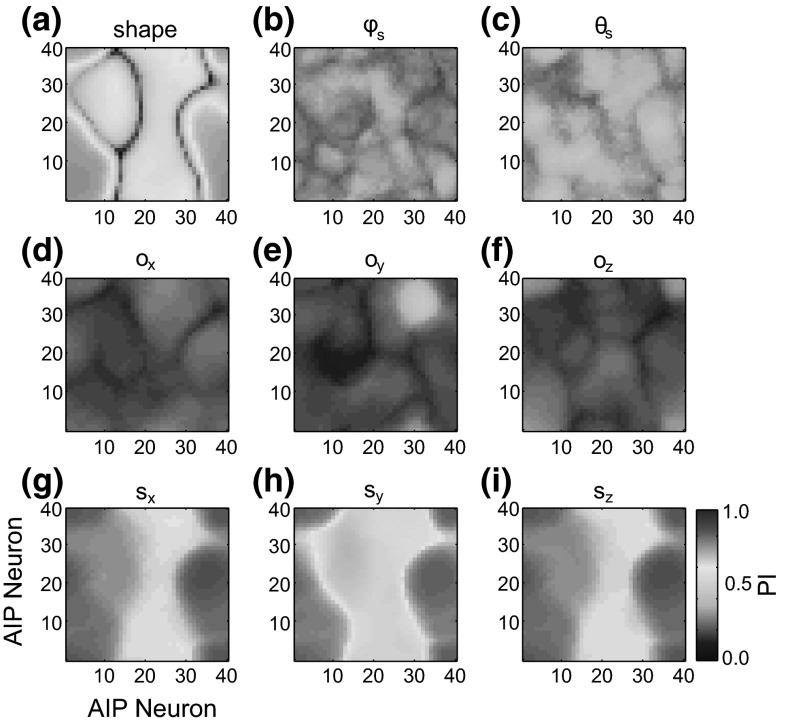
Preference index (PI) for each AIP neuron for different object features after training. **a** Shape, **b** shoulder-centered direction ${\varphi }_{s}$, **c** shoulder-centered direction $\theta _\mathrm{s}$, **d** orientation, ${o}_{x}$, **e** orientation, ${o}_{y}$, **f** orientation ${o}_{z}$, **g** size, ${s}_{x}$, **h** size, ${s}_{y}$, **i** size, $s_{z}$

**Fig. 11:**
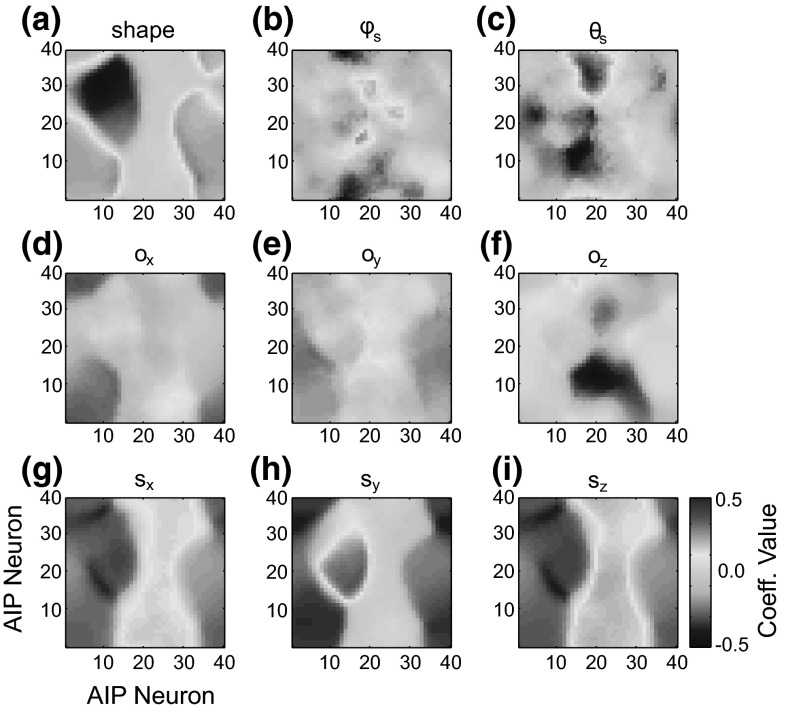
Value of each fitted linear coefficient for each AIP neuron after training. **a** Shape, **b** shoulder-centered direction ${\varphi }_\mathrm{s}$, **c** shoulder-centered direction ${\theta }_\mathrm{s}$, **d** orientation, ${o}_{x}$, **e** orientation, ${o}_{y}$, **f** orientation ${o}_{z}$, **g** size, ${s}_{x}$, **h** size, ${s}_{y}$, **i** size, ${s}_{z}$

At the end of training, we computed the PI for each AIP neuron for object shape, shoulder-centered direction $({\varphi }_{s},\, {\theta }_\mathrm{s})$, orientation $({o}_{x},\, {o}_{y},\, {o}_{z})$, and size $({o}_{x},\, {o}_{y},\, {o}_{z})$. The PI for each AIP neuron for each object feature is shown in Fig. 10. A significant portion of AIP neurons were moderately selective for object shape, position, orientation, and size. Each AIP neuron responded to a combination of these object features. We fitted the activity of each AIP neuron to a linear model using the z-scored value of each object feature as independent variables. The coefficients for each variable for all AIP neurons are shown in Fig. 11. While many neurons are highly selective for the object shape, neurons within those populations preferentially respond to a combination of different orientations, positions, and sizes.

**Fig. 12:**
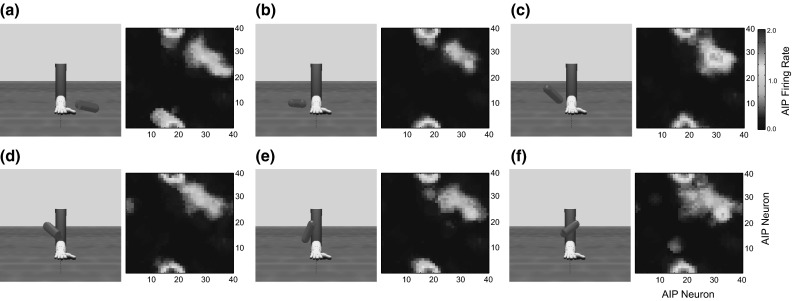
Response of each AIP neuron after training to a cylinder of the same size presented at different orientations and positions

We tested the ability of AIP representations to encode different object orientations and positions by presenting the model with a cylinder of a given size at various orientations and positions (Fig. 12). In all trials, the cylinder elicited a similar pattern of activation in AIP, but certain cells were modulated by the orientation and/or position of the object. The AIP module therefore represented not only the grasp affordance represented by the object (a power grasp in this case), but also the metric information needed to parameterize the grasp according to the object’s position and orientation.

### Grasp training

**Fig. 13:**
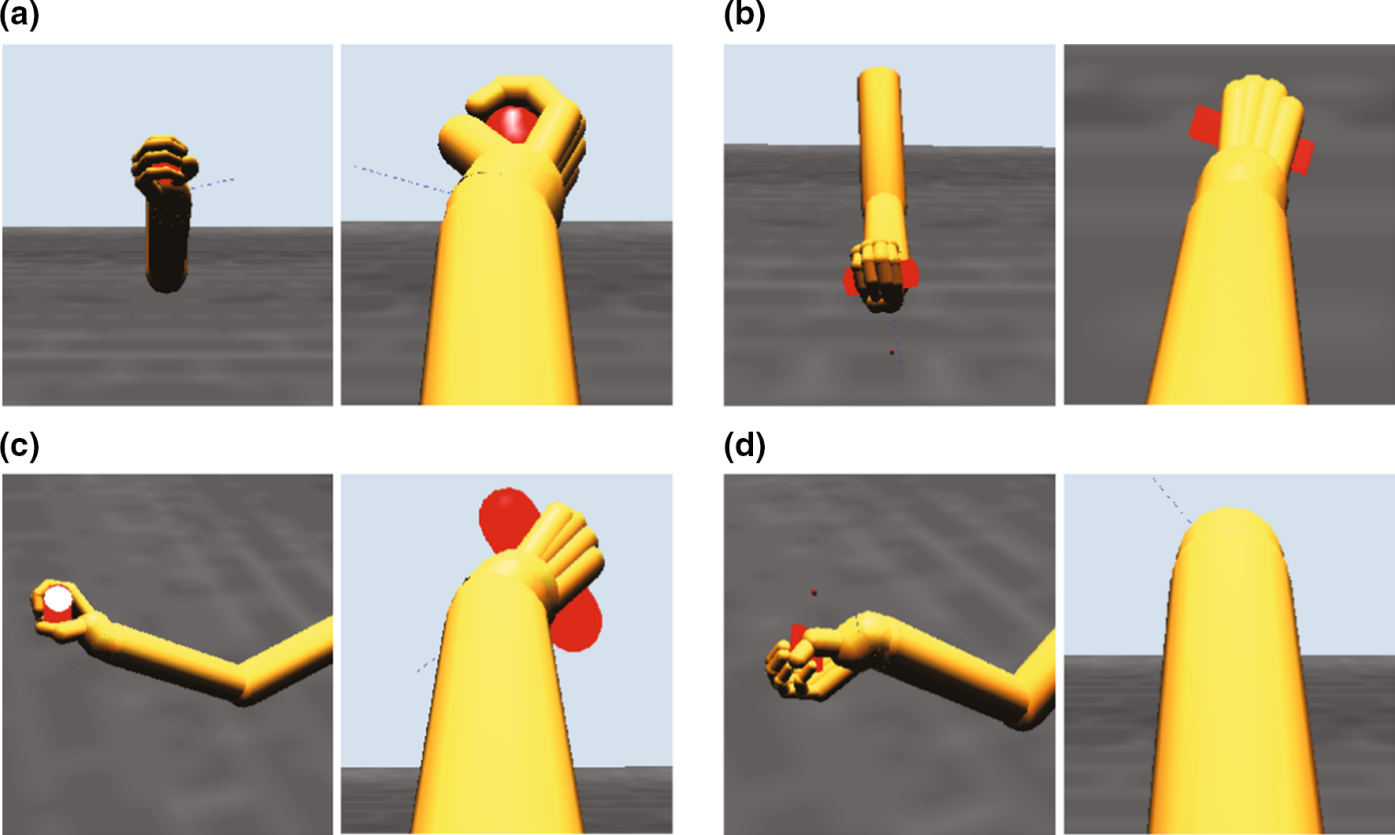
Stable power grasps generated by the model of different objects with various positions and orientations. *Each panel* shows a third-person view on the *left* and the model’s first-person view on the *right*. **a** Sphere, **b** rectangular prism, **c** cylinder, **d** plate

**Fig. 14:**
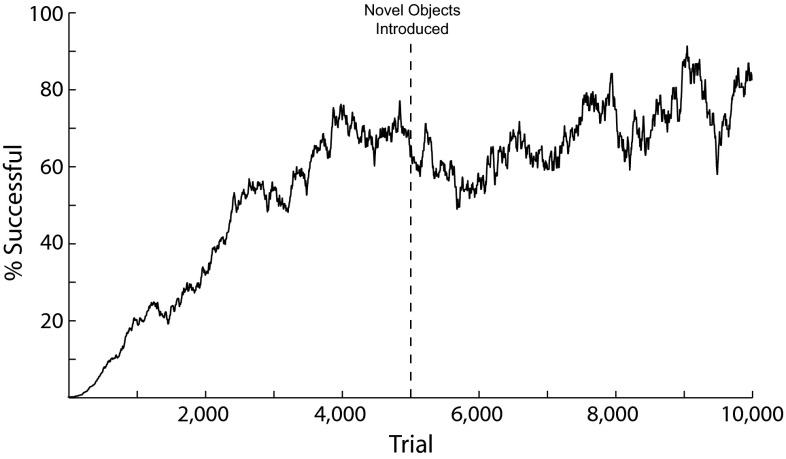
Percentage of trials resulting in a stable grasp during training

**Fig. 15:**
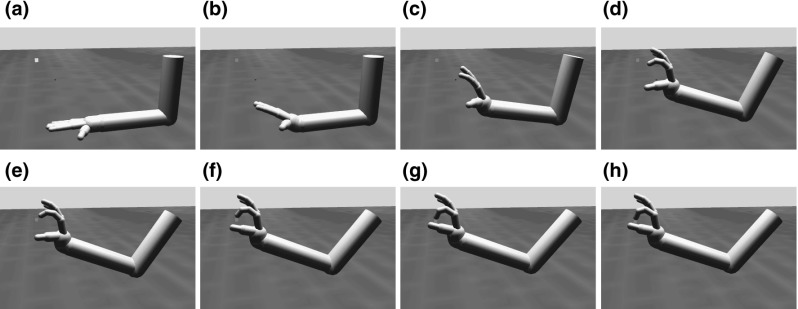
A series of frames showing the progression of a precision pinch of a small cube generated by the model. After the go signal (**b**), hand preshaping and wrist rotation has begun (**c**). The enclose phase is triggered in d and the object is first contacted in f

For each object shape and configuration, the model replicated the results of the ILGM, which used a simplified AIP model that signaled the presence, location, or orientation of an object in different simulations. As in ILGM, the learned connection weights between the AIP module and the premotor populations encoded grasp parameters most likely to result in the performance of a stable grasp. The model was able to generate stable grasps of each object tested in various positions with different orientations (Figs. 13, 15, see Online Resources 2–6 for videos of sample grasps), successfully grasping the presented object in nearly 70 % of the trials after 5000 training trials (Fig. 14). Halfway through the training period, we introduced a set of novel object trials, presenting the model with objects having the same shapes as objects it had seen before, but in new positions, orientations, and having different sizes. Performance initially decreased to approximately 50 % at 6000 trials, but then increased to nearly 80 % after 8000 trials.

ILGM showed that even without hand preshaping and virtual finger enclosure, precision pinches could result from trial-and-error reinforcement learning, although not often. We have replicated this result, but because the more realistic physics simulator we used is much less forgiving in evaluating grasp stability, we had to build the ability to preshape the hand for a precision pinch into the model. In spite of this limitation, we have shown that it is possible to learn appropriate affordance representations and motor parameters for precision pinches without feedback-based control. We therefore believe that the development of precision pinching coincides with the development of feedback-based, skilled grasping (see Sect. 4). A sequence of frames from a precision pinch generated by the model during training is shown in Fig. 15 (see Online Resource 2 for the video). Hand preshaping and wrist rotation begins shortly after object presentation (Fig. 15c), and the enclosure phase is triggered once the grasp reaches its maximal aperture (Fig. 15d). The forefinger first makes contact with the object (Fig. 15f), and the thumb contacts the other side of the object’s surface (Fig. 15g). The opposition axis between the thumb and forefinger was aligned close enough to stabilize the object, aided by friction, and the resulting grasp is judged as stable (Fig. 15h).

**Fig. 16:**
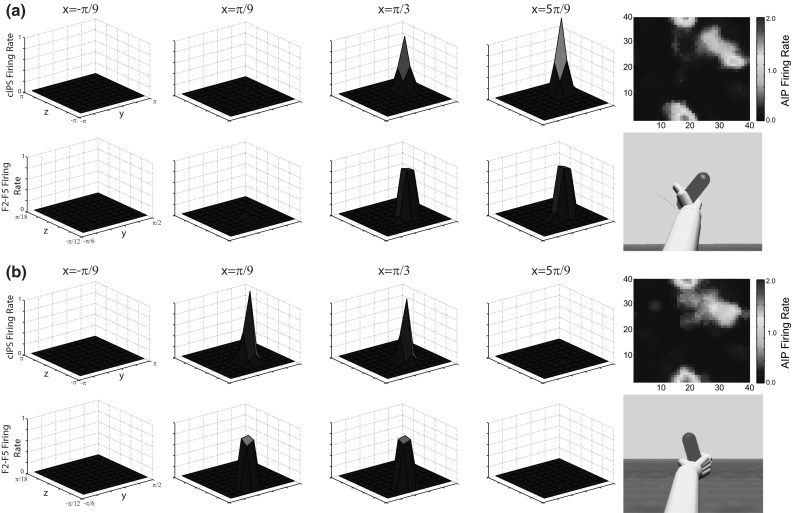
Top row of each panel shows (from *left* to *right*) firing rates of slices of the cIPS AOS cylinder population at ${x}=-\pi /9,\, \pi /9,\, \pi /3,\, 5\pi /9$ and the AIP population. The *bottom row* of each panel shows (from *left* to *right*) firing rates of slices of the F2/F5 population at $\hbox {x}=-\pi /9,\, \pi /9,\, \pi /3,\, 5\pi /9$ and the resulting grasp. **a** Network activity and resulting grasp in response to a cylinder rotated 60 degrees about the *x* axis. **b** Network activity and resulting grasp in response to a cylinder rotated 120 degrees about the *x*-axis. Activity in other populations was not significantly different during each grasp and is therefore not shown

As demonstrated above, the learned AIP representation preserved those features represented in cIPS and V6A/MIP that are essential for programming the grasp, allowing the model to successfully grasp objects in various orientations and positions. Activity in cIPS, AIP, and premotor cortex is shown in Fig. 16 during two grasps of the same size cylinder at the same location (sometimes the object is displaced by the hand after grasping), but with different orientations. The cIPS AOS cylinder population encodes the three-dimensional orientation of the main axis of the cylinder as a two-dimensional population code. Based on this representation and those in the other cIPS populations and V6A/MIP, the AIP module forms a distributed representation of combinations of the object’s features that are important for grasping. AIP neurons selective for the object shape and/or position are active during each grasp trial, resulting in highly similar patterns of activity in AIP. However, there are some AIP neurons selective for the orientation of the object, and this causes the patterns of AIP activity to be slightly different depending on the object’s orientation in each trial. These neurons bias motor parameter selection in the premotor populations, resulting in the selection of parameters appropriate for the current object’s orientation. The differences in F2–F5 activity encode the different wrist rotations that must be used to successfully grasp the cylinder at each orientation.

**Fig. 17:**
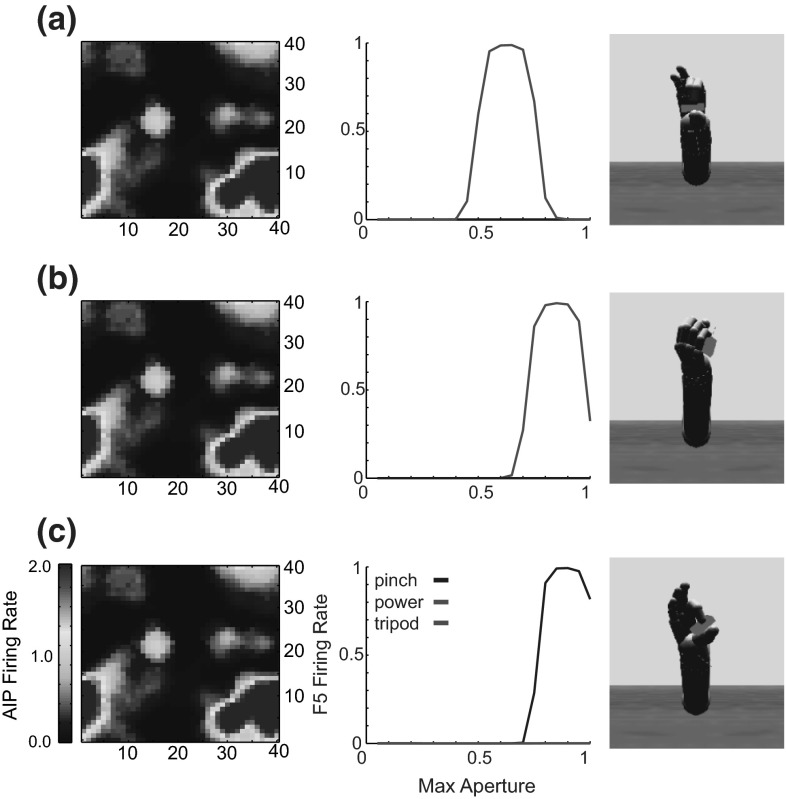
Firing rates of neurons in AIP (*left column*) and F5 (*middle column*) while grasping a plate with a tripod grasp (**a**), power grasp (**b**), and precision pinch (**c**)

In this model, F5 neurons encode grasp types, since their activity is decoded in order to perform the grasp. In contrast, AIP neurons come to represent affordances—combinations of object features that signal the possibility for grasping. A comparison of AIP and F5 activity while grasping the same object with different types of grasps is shown in Fig. 17. Each row shows AIP and F5 activity while grasping the same object with three different types of grasps. The AIP representation is almost exactly the same during each grasp, but the F5 activation patterns are completely different. AIP neurons in this model therefore do not specify the type of grasp, but represent an affordance that can be acted on using several types of grasps.

**Fig. 18:**
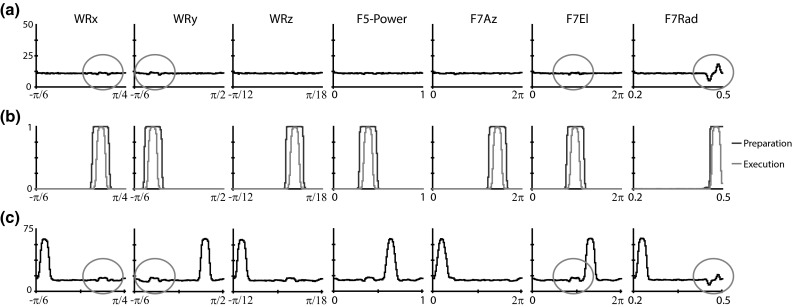
An example of the generalization abilities of ILGA. **a** Input to each premotor population during the first trial with a novel object. The *circles highlight* biases based on previous successful grasps of similar objects. **b** Premotor signal and execution subpopulation activity on the first trial with the novel object. **c** Input to each premotor population after 35 trials with the novel object

Because of its learned affordance representation, ILGA can generalize learned grasp plans to novel objects. After training, the model was presented with a novel object at a similar orientation and position to those in the training set. Figure 18a shows the input to each premotor population during the first trial with the novel object, and Fig. 18b shows the resulting premotor population activity in that trial. The circles denote regions of grasp parameter space where the model was biased toward selecting grasp parameters that had proved successful with similar objects during training. These parameters resulted in successful grasps of the novel object in some proportion of trials, but after 35 trials, a better strategy was found, demonstrated by the shifts in the peaks of input activity to each premotor population in Fig. 18c.

## Discussion

The main computational ingredients in ILGA and their contributions to the main results of our simulations are summarized in Table 1. We have shown not only that the current model explains the development of F5 canonical neurons controlling grasping, as did ILGM, but that it also gives an account of the development of visual neurons in area AIP. In our simulations, highly shape-specific neurons developed in this region as the result of unsupervised learning before grasps were even attempted. This specificity was maintained even as grasps were performed. The result was a mixture of AIP neurons that only fire for particular shapes and those that are activated by combinations of features representing grasp affordances. This allows the model to respond to novel objects in positions and orientations similar to ones it has already successfully grasped.

**Table 1:** Summary of ILGA components and results

Component	Result
Object feature population codes	Aids generalization between objects with slightly different features (i.e., objects of a similar size or orientation)
Self-organizing map (SOM)	Aids generalization between objects with similar combinations of features and novel objects with feature combinations similar to those seen before
Modulation of SOM learning rate by reinforcement	Causes SOM to preferentially represent feature combinations of successfully grasped objects
Dynamic neural fields (DNFs)	Allow selection of motor parameters based on input strength
Noise in DNF activity	Promotes exploration of parameter space
Learned connection weights between AIP and premotor DNFs	Bias the selection of reach and grasp parameters to successfully grasp object
Learned connection weights between premotor DNFs	Bias the selection of reach and grasp parameters that depend on values of other parameters (i.e., the appropriate wrist rotation depends on the chosen reach offset)
Fixed connection weights between V6a/MIP and premotor	Allow accurate reaches. Future versions could change with learning to simulate reach learning
Dynamic movement primitives (DMPs)	Implement reach trajectory planning in a way that is easily extended to handle more complex trajectories

Parietal representations in both ILGA and its precursor, ILGM, use population codes to represent object features, but ILGA represents multiple object features at once and combines them into a representation of a grasp affordance. ILGM’s premotor module uses a probabilistic coding followed by a rewriting of activity as a population code, while ILGA uses a more realistic noisy WTA process. In ILGM the wrist rotation, object-centered reach offset and hand enclosure rate are selected by the premotor module, but ILGA comes closer to FARS in including the grasp type and maximum aperture in addition to wrist rotation and object-centered reach offset.

Oztop et al. (2006) presented a model of AIP related to ILGM and ILGA. Like ILGA, this model used a SOM to model visual-dominant AIP neurons. The basic result of this model was the demonstration of how AIP could extract higher-level information from simpler visual inputs and map them onto hand postures resulting in stable grasps. In this sense, the results of ILGA are similar, showing how AIP can combine simpler visual input into higher-level affordance representations and map them onto motor parameters that will result in stable grasps. However, Oztop et al. (2006) used backpropagation, a biologically implausible learning rule, to shape the connections between AIP and F5, while ILGA uses reinforcement learning. The inputs and outputs to Oztop et al.’s (2006) model are also less biologically plausible than in ILGA. In their model, the input to AIP was a depth map and the F5 representation was set of hand joint angles. This is inconsistent with the neurophysiological data showing representation of object geometric properties in cIPS (Sakata et al. 1998) and its projection to AIP (Nakamura et al. 2001), as well as data showing that F5 neurons are tuned to a particular grasp rather than the specific posture of the hand at any point during the grasp (Umilta et al. 2007).

### Skilled grasping

Normally, grasping is controlled in a feedforward manner (Santello et al. 2002; Winges et al. 2003); however, several studies have found online feedback-based corrections of the hand shape relative to the object in the later part of the movement (Schettino et al. 2003; Nataraj et al. 2014) especially during object perturbations (Gritsenko et al. 2009) and as a compensatory strategy after stroke (Raghavan et al. 2010). This indicates that humans are capable of both types of grasp control and can switch between them based on task demands and availability of sensory information (Hoff and Arbib 1993; Gritsenko et al. 2009). Indeed, transient inactivation of human area AIP using TMS causes deficits in error correction during online control of grasping (Tunik et al. 2005). While the current model accounts for the development of visually dominant AIP neurons, it does not include motor and visuomotor neurons. It is thought that these neurons complete a feedback loop between AIP and area F5 that is used to perform feedback-based control of grasping and other manual actions. In order for this model of open loop control of infant grasping to include feedback control to guide the fingertips to the object’s surface, two extensions are required. The first is a region to represent patches of an object’s surface to serve as targets to bring the fingertips to, and the second is an *inverse* kinematics model of the hand and wrist to bring the desired virtual fingers to these targets. Introduction of these features to the model would both increase the granularity of object representation and improve fine control of the hand, expanding the range of possible grasp affordances that can be extracted and acted upon.

Since the orientation of the surface patches matters in programming a grasp, a candidate region for the object surface patch representation would be cIPS since surface-orientation-selective cells have been found there (Sakata et al. 1997). To make the target surface representation invariant to object location, it may be represented in an object-centered reference frame. Although such an organization has not been reported in cIPS, this may be due to a lack of experiments using eye tracking to control for gaze position with respect to the center of the object. A potential problem with this idea is that cIPS neurons have only been found to respond to visible surfaces, whereas fingertips may potentially contact a surface on the opposite side of an object during a grasp. The location of potential target surfaces on the opposite side of a visible object must therefore be inferred from the 2 1/2-dimensional sketch of the object provided by cIPS.

The current model uses an inverse kinematics model of the arm to convert target wrist locations into target joint angles for the shoulder and elbow. In order to perform more precise and dextrous grasp and manipulation tasks, the extended model must include an inverse kinematics model of the arm and hand that can convert target locations for any combination of virtual fingers into target joint angles for the shoulder, elbow, wrist, thumb, and finger joints. ILGA uses the pseudo-inverse of the Jacobian matrix for computing inverse reach kinematics, requiring a $3\times 4$ matrix (4 controlled degrees of freedom, 3-dimensional wrist position). If the extended model used the same method, this would require multiple Jacobian matrices, one for each combination of virtual fingers and mapping to real fingers. Assuming that only two fingers will contact the object, each Jacobian would therefore be a $6\times 22$ matrix (22 controlled DOFs, and two 3-dimensional virtual finger positions). Since multiple combinations of virtual fingers, mappings to real fingers, and contact points are possible, this method may not be tractable.

A model like ILGA could provide the scaffolding for such a model by generating a range of stable grasps that can be used to learn the prediction of unseen target surfaces. Given a stable grasp, the haptic feedback from finger and hand contacts with the object can be used to calculate an object-centered representation of the location of these contact points using the location of the object (represented in V6A/MIP) and the posture of the harm and hand. These contact point locations could then be used as a training signal for a network that predicts the location of target surfaces given the representation of visible surfaces in cIPS. Such a model would then develop AIP cells that are responsive to the affordances characterized by these combinations of surface patches. Computing the positions of hand–object contact points given the arm/hand posture requires a *forward* kinematics model of the entire arm and hand including shoulder, elbow, wrist, thumb, and finger joints.

The extended model therefore requires a set of inverse/forward model pairs for the entire arm/hand. The models could be learned using multiple model-based reinforcement learning (MMRL, Doya et al. 2002) during the ILGA training period. MMRL is a method of using reinforcement learning and predictor error to learn multiple inverse/forward model pairs, and has been formulated in both discrete and continuous time and state cases. This architecture is composed of multiple modules containing inverse/forward model pairs that compete to learn new tasks, with certain modules becoming specialized for different tasks with repeated training. These models could be learned offline while ILGA training progresses, until the prediction errors of the forward models become small enough to allow their associated inverse models to control the arm and hand. At this point, the forward model pairs would continue to compete for control of the arm and hand and become specialized for controlling different types of grasps.

### Context-dependent grasps

At least two studies show that infants 1–2 years old selectively modify their actions based on future planned actions. McCarty et al. (1999) demonstrated that 9- and 14-month-old infants grasp a spoon with their preferred hand, regardless of its orientation and whether the next action was to bring the spoon to the mouth or another location. At 19 months, infants had learned to coordinate hand selection with the action goal and the spoon’s orientation in order to facilitate the smooth execution of the next action. Claxton et al. (2003) measured the arm kinematics of 10-month-old infants reaching for a ball and then either throwing it or fitting it down a tube. They found that the reach to the ball was faster if they then intend to throw it. Both of these studies suggest that infants preplan segments of compound actions at some level. This point brings to light a shortcoming in both the ILGM and the ILGA models. Both of these models use some evaluation of the stability of the grasp as the metric for reinforcement. A more realistic model would use success in a subsequent action with the object such as throwing or placing as the grasp reinforcement criterion. This would require some representation of the task or goal with the ability to bias selection of grasp targets and affordance representations to perform grasps appropriate for the planned action.

In the original FARS model, working memory and task-specific associations in prefrontal cortex bias grasp execution by modulating the grasp selection in F5 such that those grasps appropriate for the current task are selected. Using synthetic brain imaging, a method to compare global model activity with PET or fMRI data, Arbib et al. (2002) showed that a projection from PFC to AIP rather than F5 better explained human PET data. A projection from PFC to AIP had not been reported at that time, but this prediction was later validated both anatomically (Borra et al. 2007) and neurophysiologically (Baumann et al. 2009). This could be included in ILGA by the addition of a prefrontal cortex module encoding the task context with projections to the AIP module that are also modifiable through reinforcement. Thus, task representations in prefrontal cortex would presumably become associated with the affordances and actions that lead to reward. Such a simulation could shed insight into the interplay between cognitive and motor development by examining the operation of the model without or without a pretrained motor system or prefrontal cortex. It could be that associative signals from an already trained prefrontal cortex could interfere with the normal development of the parieto-premotor connection weights.

The issue of context-dependent grasps also requires a more sophisticated mechanism for representing multiple affordances. We suggest that the dorsal visual stream analyzes the affordances of the components of complex objects, but in general, objects are not made of spheres and rectangular solids. Rather, they may have diverse shapes, yet the developing brain learns to embed an opposition space in them that affords a stable grasp. There is additionally a binding problem here if there are multiple affordances, which may require encoding a “focus of attention” to give an approximate localization of the affordance with learning refining this to the localization of the grasp axis to guide the grasp-plan-specific guidance of reaching.

### Predictions

Using biologically plausible learning rules and inputs, we have shown that ILGA can learn to represent affordances for grasping and to select motor parameters appropriate to act on them. This model makes several testable predictions concerning (a) the encoding of object features in area AIP, (b) shifts in AIP activation during learning, and (c) the existence of an object-centered spatial representation for reach-to-grasp movements.

ILGA reproduces experimental data showing that many AIP neurons are moderately object shape selective, showing responses to multiple objects (Murata et al. 2000). Since ILGA is a developmental model, it allows us to go beyond available experimental data and predict that AIP neurons are initially selective for various object features and become shape selective early in development, before grasping has developed. In ILGA, the global reinforcement signal elicited by successful grasps modulates the rate of unsupervised learning occurring in AIP. This causes neurons in AIP to preferentially encode features of objects that can be successfully grasped, resulting in a representation of grasp affordances rather than strictly geometric features. To our knowledge, no studies have looked for shifts in AIP activation during the course of grasp learning. This model predicts that if grasps of certain objects are disrupted (through local muscimol injection or physical perturbation), AIP cell selectivity will shift, with more cortical representation eventually given to the features of other objects that are successfully grasped. For example, the selectivity of any AIP neurons that prefer round, elongated objects should shift over many trials if grasps of cylinders are repeatedly disrupted by spatial perturbing the target object.

While we are not aware of any data showing the existence of an object-centered reference frame for reaching, we found it necessary to use such a representation in order to plan the direction of the hand’s approach to the object. One possible reason that such a representation has not been found is that most experiments use either a pure reaching, wrist rotation, or naturalistic grasping task. Once ILGA has been trained, the reach offset direction is highly correlated with the wrist rotation so that the hand will approach the object with the correct orientation for grasping. Therefore, selectivity for object-centered offset directions cannot be experimentally demonstrated without trials in which the offset direction is held constant while the wrist rotation is varied. This is similar to the situation in the interpretation of motor cortex activity, where it has been shown that intrinsic and extrinsic and kinematic and kinetic variables are highly correlated during a commonly used experimental reaching task (Chan and Moran 2006). In order to determine whether a region encodes the object-centered reach offset independent of wrist rotation, a reaching task must be used in which the subject must reach to a target object from different directions with varying wrist orientations. On the basis of its object-centered representation for saccades, and arm-related activity in its ventral portion, we predict an object-centered spatial representation in ventral F7.

### Related models

Most related grasping models focus on learning inverse kinematics for the hand, selecting contact points on the object’s surface, and developing feedback-based control of the hand. Most models plan the grasp in terms of kinematics, but at least one model stresses control of grasp forces. While many models use trial-and-error learning, they are not developmental models like ILGM and ILGA in the sense that they begin learning at a phase corresponding to grasping development in infants 9 months and older. Some models are based on neurophysiological data, but many are built purely using machine learning and robotics techniques.

Two models that stress learning inverse kinematics transformations for the hand are those of Molina-Vilaplana et al. (2007), Rezzoug and Gorce (2003) and Gorce and Rezzoug (2004). Molina-Vilaplana et al.’s (2007) model first learns the inverse kinematics functions of the fingers and then learns to associate object properties with grasp postures (a function they relate to AIP/F5 functionality). The model first learns inverse kinematics for the thumb, index, and middle fingers so that it knows the relationships between finger motor commands and their sensory consequences (proprioceptive and visual), and then learns to associate object features with grasp postures with a local network called GRASP. The input to GRASP is a 7-dimensional vector encoding object shape (cube, sphere, or cylinder), object dimensions, and whether to grasp with two or three fingers. Similarly, Rezzoug and Gorce (Rezzoug and Gorce 2003; Gorce and Rezzoug 2004) have a model with a modular architecture that first learns inverse kinematics for the fingers using backpropagation and then learns hand configuration for grasping using reinforcement. The Fingers Configuration Neural Network (FCNN) learns finger postures given the desired position of the fingertip.

Several models plan grasps in terms of finger contact points on the object. Molina-Vilaplana et al. (2007) use heuristics to select contact points on the object surface for the fingers. Rezzoug and Gorce’s (Rezzoug and Gorce 2003; Gorce and Rezzoug 2004) model uses a Hand Configuration Neural Network (HCNN) that learns contact locations for each finger. Kamon et al. (1996) split the problem of grasp learning into two problems: choosing grasping points and predicting the quality of the grasp. Each grasp type has a certain set of location and quality parameters used to select grasp locations and predict quality, which are supplied beforehand as task-specific knowledge. Grasp quality is predicted by the angles between the fingers and the surface normals at the contact points, and the distance between the opposition axis and the center of mass. They suggest that the grasp specification and evaluation modules run in an alternating manner until a suitable grasp is selected. Faldella et al. (1993) present an interesting model where a neural mechanism for matching object geometry to hand shape interacts with a symbolic rule-based expert system. The symbolic system performs some geometric analyses such as identifying curvature type, selects candidate hand contact positions, identifies symmetric situations (to reduce neural module input), and ranks the selected grasp according to task constraints. Multilayer perceptrons trained using backpropagation were used to determine potential grasps based on geometric information.

For Grupen and Coelho (2000), grasping is primarily a force domain task, emphasizing *force* closure around an object over *form* (of the hand) closure. Their model used closed loop control, utilizing tactile feedback to reposition contact forces based on models of interaction between the contacts and object surface. A Markov decision process (MDP) framework is used to select a sequence of controllers to maximize object stability without knowing the object’s identity, geometry, or pose. This model is complementary to those that focus on visual-based kinematic grasp planning in that it learns to use haptic feedback in order to reposition contact forces to stabilize the object. Including such a mechanism in a model like ILGA would increase the number of successful grasps early in training and may provide a way to adjust grasp plans that lead to initially unstable grasps that can be subsequently stabilized with corrective hand movements.

The Two Route, Prefrontal Instruction, Competition of Affordances, Language Simulation (TRoPICALS) model is a neural network model similar to FARS which includes the influence of the dorsal and ventral stream in affordance selection and grasp planning (Caligiore et al. 2010). Like ILGA, TRoPICALS utilizes SOMs, winner-take-all processes, and a staged learning approach to learn to represent object shapes and motor parameters appropriate for grasping them. While ILGA does not include the ventral visual stream, TRoPICALS includes the influence of language and ventral stream recognition of object identity on affordance selection and is able to reproduce the pattern of reaction times shown by human subjects when presented with conflicting grasp instructions and affordances. However, ILGA uses a more complex simulation environment, captures the emergence of affordance representations in AIP, and addresses the linkage between reaching and grasping in ways that TRoPICALS does not.

Each of these models addresses some aspect of grasping that is neglected in this model. In ILGA, only the inverse kinematics for the arm is used to plan reaching movements, while grasping movements are controlled by rotating the wrist and preshaping then enclosing the fingers. Grasping is controlled in an open loop manner without a notion of target contact points on the object surface, and kinetics is handled by PD controllers that are not concerned with balancing force application along an opposition axis. However, ILGA is more biologically plausible in that it uses neural representations based on neurophysiological data, modules with connectivity constrained by anatomical data, and biologically inspired learning rules. ILGA could be extended to a more complete model by including aspects of these other models such as inverse kinematics for the hand and feedback-based grasp control (described in Skilled grasping, above), using visual feedback for the control of the hand, and utilizing haptic feedback for corrective movements.

Integration of ILGA with models of the primate mirror system could provide the system with a feedback signal for skilled grasping. The neurons in the AIP module of ILGA correspond to visual-dominant neurons in monkey area AIP and project to the F5 module, but do not receive reciprocal connections from F5. In reality, area AIP also contains neurons classified as visuomotor and motor dominant and receives feedback from F5 (Sakata et al. 1995). Motor-dominant cells respond during grasping in the light and the dark, while visual-dominant cells respond only during object fixation and grasping in the light and visuomotor cells respond during object fixation and grasping in the dark but fire most strongly during grasping in the light. Sakata et al. (1995) offer a conceptual model of feedback-based grasping in which F5 canonical neurons provide AIP motor-dominant neurons with a copy of the grasp motor command, which then pass this signal to AIP visuomotor neurons which combine this information with information from AIP visual-dominant neurons and project back to F5. In this way, if the ongoing grasp does not match the encoded affordance, the grasp plan in F5 is modified or aborted. AIP visual-dominant neurons are classified into object-type neurons that fire during object fixation, and non-object-type neurons that fire during grasping in the light but not object fixation and may respond to the sight of the hand during the grasp. Non-object-type neurons are seldom mentioned in discussions of AIP, but make up half of visual-dominant neurons in the region (Sakata et al. 1995). Interestingly, their existence fits with the hypothesis outlined by Oztop and Arbib (2002) that F5 mirror neurons evolved to provide visual feedback of the shape of the hand relative to the object’s affordances. We suggest that non-object-type AIP neurons obtain their properties by projections from F5 mirror neurons, and that these projections are used for visual feedback-based control of grasping. It has been shown that reversible inactivation of F5 mirror neurons by muscimol injection in the cortical convexity of the arcuate sulcus results in slower, clumsy grasps (Fogassi et al. 2001), consistent with the idea of F5 mirror neurons playing a role in providing visual feedback. Our conceptual model predicts that muscimol injection in the cortical convexity of F5 will abolish the response of non-object-type AIP neurons to the sight of the grasp.

In addition to reversible inactivation of mirror neurons, Fogassi et al. (2001) tested the effects of muscimol injection in the bank of the arcuate sulcus where most F5 canonical neurons are located. In this case, the hand preshape was impaired but monkeys were still able to grasp the object by contacting it and then making appropriate corrective movements using tactile feedback. This seems similar to the process modeled by Grupen and Coelho (2000) in which haptic feedback is used to reposition contact forces. The fact that corrective movements can still be made after F5 inactivation suggests that they are not based on F5 activity and may be implemented by the direct projection included in the FARS model from the primary somatosensory area S1 to the primary motor cortex.

#### Future directions

ILGA combines grasp learning ability with a mature reaching system that is smooth and accurate. However, in infants, reaching and grasping develop simultaneously. By allowing the development of simultaneously maturing reach and grasp systems to influence each other, future versions of the model may be able to address data showing how the reach and grasp movements are coordinated based on the object to be grasped (Supuk et al. 2005) or the distal goal of the movement (i.e., grasping to lift versus to place, Ansuini et al. 2006).

We have used as realistic simulation of the arm, hand, and object as possible, within the limitations of our simulation engine, in order to facilitate translation of ILGA to a robotic platform. However, several issues would need to be addressed for this transition. To simplify the task, we did not include gravity, so that objects had mass, but no weight. This required the model to preshape the hand and enclose it around the object to stabilize it, but did not require the arm to counteract the weight of the object. Additionally, we did not simulate objects made of different materials. All of our objects had the same friction coefficient and therefore a robotic implementation of ILGA would require the computation of the appropriate contact forces for objects of different types.

ILGA represents reach and grasp rotation parameters using Euler angles. While this representation is more compact than rotation matrices, for example, it suffers from several problems such as composition difficulty (Pastor and Righetti 2011) and singularities which can cause gimbal lock (Feix and Romero 2013). Quaternions use one more parameter than Euclidean angles, but do not suffer from these problems while still having lower dimensionality than rotation matrices (Feix and Romero 2013). It is not known what representation the brain uses to represent joint rotation, but future versions of ILGA could explore the use of quaternion representations to avoid the issues caused by Euler angles and reduce dimensionality.

## Conclusion

ILGA is the only developmental model of grasping to date that simultaneously learns to extract affordances from object features and select motor parameters to successfully grasp them. We have shown that the model develops distributed representations in area AIP similar to those reported in the experimental literature and can use these representations to generalize grasp plans to objects of varying sizes and at different orientations and positions. Finally, we presented several neurophysiologically testable predictions made by the model and discussed ways in which it could be extended to handle context-dependent grasping of complex objects and skilled manipulation.

## Electronic supplementary material

Supplementary material 1 (docx 31 KB)

Supplementary material 2 (avi 579 KB)

Supplementary material 3 (avi 596 KB)

Supplementary material 4 (avi 629 KB)

Supplementary material 5 (avi 586 KB)

Supplementary material 6 (avi 607 KB)
