# Supplementary material for: Learning to grasp and extract affordances: the Integrated Learning of Grasps and Affordances (ILGA) model
Source: Biol Cybern. 2015 Nov 19;109:639–69. doi: 10.1007/s00422-015-0666-2 (PMC4656720; doi:10.1007/s00422-015-0666-2)
Supplement: Supplementary file 1 — Supplementary material 1 (docx 31 KB) [file 422_2015_666_MOESM1_ESM.docx]

Learning to Grasp and Extract Affordances: The Integrated Learning of Grasps and Affordances (ILGA) Model

James Bonaiuto1,2,3, Michael A. Arbib2,3,4

1Institute of Neurology, University College London, London

2Neuroscience Program, 3USC Brain Project, 4Computer Science Department, University of Southern California, Los Angeles, CA, USA

bonaiuto@usc.edu

## Supplementary Material

Table 1: Parameters of the arm/hand model.

| **Joint** | **Parameter** | **Description** | **Value** |
| --- | --- | --- | --- |
| Shoulder | *θ_x_* | Angle limits in x-axis | [-π/2 π/2] |
|  | *θ_y_* | Angle limits in y-axis | [-π/4 π/4] |
|  | *θ_z_* | Angle limits in z-axis | [-π/2 π/2] |
|  | *p_x_* | Gain parameter for PD controller in x-axis | 100000 |
|  | *d_x_* | Damping parameter for PD controller in x-axis | 1000000 |
|  | *p_y_* | Gain parameter for PD controller in y-axis | 30000 |
|  | *d_y_* | Damping parameter for PD controller in y-axis | 1000000 |
|  | *p_z_* | Gain parameter for PD controller in z-axis | 30000 |
| Elbow | *θ_x_* | Angle limits | [-π/4 π/4] |
|  | *p_x_* | Gain parameter for PD controller in x-axis | 30000 |
|  | *d_x_* | Damping parameter for PD controller in x-axis | 1000000 |
| Wrist | *θ_x_* | Angle limits in x-axis | [-π/6 π/4] |
|  | *θ_y_* | Angle limits in y-axis | [-π/6 π/2] |
|  | *θ_z_* | Angle limits in z-axis | [-π/12 π/18] |
|  | *p_x_* | Gain parameter for PD controller in x-axis | 7000 |
|  | *d_x_* | Damping parameter for PD controller in x-axis | 1000000 |
|  | *p_y_* | Gain parameter for PD controller in y-axis | 8000 |
|  | *d_y_* | Damping parameter for PD controller in y-axis | 1000000 |
|  | *p_z_* | Gain parameter for PD controller in z-axis | 5000 |
|  | *d_z_* | Damping parameter for PD controller in z-axis | 800000 |
| Thumb – carpometacarpal joint | *θ_x_* | Angle limits in x-axis | [0 π/2] |
|  | *θ_z_* | Angle limits in z-axis | [-π/6 0] |
|  | *p_x_* | Gain parameter for PD controller in x-axis | 5000 |
|  | *d_x_* | Damping parameter for PD controller in x-axis | 320000 |
|  | *p_z_* | Gain parameter for PD controller in z-axis | 5000 |
|  | *d_z_* | Damping parameter for PD controller in z-axis | 320000 |
| Thumb - metacarpophalangeal joint | *θ_x_* | Angle limits in x-axis | [0 π/2] |
|  | *p_x_* | Gain parameter for PD controller in x-axis | 5000 |
|  | *d_x_* | Damping parameter for PD controller in x-axis | 240000 |
| Index finger – metacarpophalangeal joint | *θ_x_* | Angle limits in x-axis | [0 π/2] |
|  | *p_x_* | Gain parameter for PD controller in x-axis | 5000 |
|  | *d_x_* | Damping parameter for PD controller in x-axis | 350000 |
| Index finger – proximal interphalangeal joint | *θ_x_* | Angle limits in x-axis | [0 π/2] |
|  | *p_x_* | Gain parameter for PD controller in x-axis | 5000 |
|  | *d_x_* | Damping parameter for PD controller in x-axis | 320000 |
| Index finger – distal interphalangeal joint | *θ_x_* | Angle limits in x-axis | [0 π/2] |
|  | *p_x_* | Gain parameter for PD controller in x-axis | 5000 |
|  | *d_x_* | Damping parameter for PD controller in x-axis | 240000 |
| Middle finger – metacarpophalangeal joint | *θ_x_* | Angle limits in x-axis | [0 π/2] |
|  | *p_x_* | Gain parameter for PD controller in x-axis | 5000 |
|  | *d_x_* | Damping parameter for PD controller in x-axis | 350000 |
| Middle finger – proximal interphalangeal joint | *θ_x_* | Angle limits in x-axis | [0 π/2] |
|  | *p_x_* | Gain parameter for PD controller in x-axis | 5000 |
|  | *d_x_* | Damping parameter for PD controller in x-axis | 320000 |
| Middle finger – distal phalangeal joint | *θ_x_* | Angle limits in x-axis | [0 π/2] |
|  | *p_x_* | Gain parameter for PD controller in x-axis | 5000 |
|  | *d_x_* | Damping parameter for PD controller in x-axis | 240000 |
| Ring finger – metacarpophalangeal joint | *θ_x_* | Angle limits in x-axis | [0 π/2] |
|  | *p_x_* | Gain parameter for PD controller in x-axis | 5000 |
|  | *d_x_* | Damping parameter for PD controller in x-axis | 350000 |
| Ring finger – proximal interphalangeal joint | *θ_x_* | Angle limits in x-axis | [0 π/2] |
|  | *p_x_* | Gain parameter for PD controller in x-axis | 5000 |
|  | *d_x_* | Damping parameter for PD controller in x-axis | 320000 |
| Ring finger – distal interphalangeal joint | *θ_x_* | Angle limits in x-axis | [0 π/2] |
|  | *p_x_* | Gain parameter for PD controller in x-axis | 5000 |
|  | *d_x_* | Damping parameter for PD controller in x-axis | 240000 |
| Pinky finger – metacarpophalangeal joint | *θ_x_* | Angle limits in x-axis | [0 π/2] |
|  | *p_x_* | Gain parameter for PD controller in x-axis | 5000 |
|  | *d_x_* | Damping parameter for PD controller in x-axis | 350000 |
| Pinky finger – proximal interphalangeal joint | *θ_x_* | Angle limits in x-axis | [0 π/2] |
|  | *p_x_* | Gain parameter for PD controller in x-axis | 5000 |
|  | *d_x_* | Damping parameter for PD controller in x-axis | 320000 |
| Pinky finger – distal interphalangeal joint | *θ_x_* | Angle joint limits in x-axis | [0 π/2] |
|  | *p_x_* | Gain parameter for PD controller in x-axis | 5000 |
|  | *d_x_* | Damping parameter for PD controller in x-axis | 240000 |

Table 2: Target angles for the finger and thumb joints for the preshape and enclose phase of side, precision, tripod, and power grasps.

| **Grasp Type** | **Phase** | **Digit** | **Joint** | **Target Angle** |
| --- | --- | --- | --- | --- |
| Side | preshape | index finger | metacarpophalangeal | π/2 |
|  |  |  | proximal interphalangeal | π/2 |
|  |  |  | distal interphalangeal | π/2 |
|  |  | middle finger | metacarpophalangeal | π/2 |
|  |  |  | proximal interphalangeal | π/2 |
|  |  |  | distal interphalangeal | π/2 |
|  |  | ring finger | metacarpophalangeal | π/2 |
|  |  |  | proximal interphalangeal | π/2 |
|  |  |  | distal interphalangeal | π/2 |
|  |  | pinky finger | metacarpophalangeal | π/2 |
|  |  |  | proximal interphalangeal | π/2 |
|  |  |  | distal interphalangeal | π/2 |
|  |  | thumb | carpometacarpal | [0 0] |
|  |  |  | metacarpophalangeal | $\left(1-maxAperture\right)\frac{\pi}{18}$ |
|  | enclose | thumb | carpometacarpal | [2π/5 –2π/9] |
|  |  |  | metacarpophalangeal | π/2 |
| precision | preshape | index finger | metacarpophalangeal | $\left(1-maxAperture\right)\frac{\pi}{2}$ |
|  |  |  | proximal interphalangeal | $\left(1-maxAperture\right)\frac{\pi}{2}$ |
|  |  |  | distal interphalangeal | $\left(1-maxAperture\right)\frac{\pi}{2}$ |
|  |  | thumb | carpometacarpal | [π/2 0] |
|  |  |  | metacarpophalangeal | 0 |
|  | enclose | index finger | metacarpophalangeal | π/4 |
|  |  |  | proximal interphalangeal | π/4 |
|  |  |  | distal interphalangeal | π/4 |
|  |  | thumb | carpometacarpal | [π/6 -π/9] |
|  |  |  | metacarpophalangeal | π/7 |
| tripod | preshape | index finger | metacarpophalangeal | $\left(1-maxAperture\right)\frac{\pi}{2}$ |
|  |  |  | proximal interphalangeal | $\left(1-maxAperture\right)\frac{\pi}{2}$ |
|  |  |  | distal interphalangeal | $\left(1-maxAperture\right)\frac{\pi}{2}$ |
|  |  | middle finger | metacarpophalangeal | $\left(1-maxAperture\right)\frac{\pi}{2}$ |
|  |  |  | proximal interphalangeal | $\left(1-maxAperture\right)\frac{\pi}{2}$ |
|  |  |  | distal interphalangeal | $\left(1-maxAperture\right)\frac{\pi}{2}$ |
|  |  | thumb | carpometacarpal | [π/2 0] |
|  |  |  | metacarpophalangeal | 0 |
|  | enclose | index finger | metacarpophalangeal | π/4 |
|  |  |  | proximal interphalangeal | π/4 |
|  |  |  | distal interphalangeal | π/4 |
|  |  | middle finger | metacarpophalangeal | π/4 |
|  |  |  | proximal interphalangeal | π/4 |
|  |  |  | distal interphalangeal | π/4 |
|  |  | thumb | carpometacarpal | [π/6 -π/9] |
|  |  |  | metacarpophalangeal | π/7 |
| power | preshape | index finger | metacarpophalangeal | $\left(1-maxAperture\right)\frac{\pi}{2}$ |
|  |  |  | proximal interphalangeal | $\left(1-maxAperture\right)\frac{\pi}{2}$ |
|  |  |  | distal interphalangeal | $\left(1-maxAperture\right)\frac{\pi}{2}$ |
|  |  | middle finger | metacarpophalangeal | $\left(1-maxAperture\right)\frac{\pi}{2}$ |
|  |  |  | proximal interphalangeal | $\left(1-maxAperture\right)\frac{\pi}{2}$ |
|  |  |  | distal interphalangeal | $\left(1-maxAperture\right)\frac{\pi}{2}$ |
|  |  | ring finger | metacarpophalangeal | $\left(1-maxAperture\right)\frac{\pi}{2}$ |
|  |  |  | proximal interphalangeal | $\left(1-maxAperture\right)\frac{\pi}{2}$ |
|  |  |  | distal interphalangeal | $\left(1-maxAperture\right)\frac{\pi}{2}$ |
|  |  | pinky finger | metacarpophalangeal | $\left(1-maxAperture\right)\frac{\pi}{2}$ |
|  |  |  | proximal interphalangeal | $\left(1-maxAperture\right)\frac{\pi}{2}$ |
|  |  |  | distal interphalangeal | $\left(1-maxAperture\right)\frac{\pi}{2}$ |
|  |  | thumb | carpometacarpal | [π/2 0] |
|  |  |  | metacarpophalangeal | 0 |
|  | enclose | index finger | metacarpophalangeal | π/4 |
|  |  |  | proximal interphalangeal | π/4 |
|  |  |  | distal interphalangeal | π/4 |
|  |  | middle finger | metacarpophalangeal | π/4 |
|  |  |  | proximal interphalangeal | π/4 |
|  |  |  | distal interphalangeal | π/4 |
|  |  | ring finger | metacarpophalangeal | π/4 |
|  |  |  | proximal interphalangeal | π/4 |
|  |  |  | distal interphalangeal | π/4 |
|  |  | pinky finger | metacarpophalangeal | π/4 |
|  |  |  | proximal interphalangeal | π/4 |
|  |  |  | distal interphalangeal | π/4 |
|  |  | thumb | carpometacarpal | [2π/5 –2π/9] |
|  |  |  | metacarpophalangeal | π/2 |

Table 3: Parameters of the ILGA model

| **Parameter** | **Description** | **Value** |
| --- | --- | --- |
| *N* | Size of each DNF population | 100 |
| *N_AIP_* | Size of the AIP population | 40×40 |
| *σ_DIST_* | Distance population code width | .05 |
| *ε_DIST_* | Distance noise | Randomly distributed between -.1 and .1 |
| *σ_DIR_* | Direction population code width | .05 |
| *ε_DIR_* | Direction noise | Randomly distributed between -.1 and .1 |
| *σ_cIPS_* | cIPS population code width | .05 |
| *ε_cIPS_* | CIPS noise | Randomly distributed between -.1 and .1 |
| *ε_AIP_* | AIP noise | Randomly distributed between -.1 and .1 |
| *ψ_AIP_* | Maximum initial weight for connections to AIP | 1.0 |
| *r_0_* | Initial neighborhood radius in AIP | *N*/2 |
| *λ* | Rate of decrease of learning rate and neighborhood size in AIP | 15000 |
| α_0_ | Initial learning rate in AIP | 0.25 |
| *ε_F2_* | F2 noise | Randomly distributed between -.1 and .1 |
| *GP* | Level of tonic inhibitory input to execution-related populations | 1.0 |
| *ψ_PM_* | Maximum initial weight for connections to premotor populations | 0.2 |
| *ε_F7_* | F7 noise | Randomly distributed between -.1 and .1 |
| *α_F7_* | F7 learning rate | .001 |
| *ε_F5_* | F5 noise | Randomly distributed between -.1 and .1 |
| *α_F5_* | F5 learning rate | .001 |
| *ε_WR_* | WR noise | Randomly distributed between -.1 and .1 |
| *α_WR_* | WR learning rate | .001 |
| *DA_fail_* | Negative reinforcement | -.001 |
| *DA_success_* | Positive reinforcement | .01 |
| *κ* | Grasp enclose phase distance threshold | .35 |

Table 4: DNF parameters

| DNF Dimensions | Parameter | Description | Value |
| --- | --- | --- | --- |
| 1 | *τ* | time constant | 75ms |
|  | *H* | baseline activation | -3 |
|  | **ε***_DNF_* | noise vector | Randomly distributed values between 0 and 3 |
|  | *β* | sigmoid slope | 1.5 |
|  | *u_0_* | sigmoid threshold | 0 |
|  | *w_excite_* | self-excitation | 2 |
|  | *w_inhibit_* | surround inhibition | 1 |
|  | *σ_DNF_* | weight kernel Gaussian width | 2 |
| 2 | *τ* | time constant | 150ms |
|  | *h* | baseline activation | -1 |
|  | **ε***_DNF_* | noise vector | Randomly distributed values between 0 and 1 |
|  | *β* | sigmoid slope | 4 |
|  | *u_0_* | sigmoid threshold | 0 |
|  | *w_excite_* | self-excitation | 1.4 |
|  | *w_inhibit_* | surround inhibition | 1.25 |
|  | *σ_DNF_* | weight kernel Gaussian width | 3 |
| 3 | *τ* | time constant | 150ms |
|  | *h* | baseline activation | -1 |
|  | **ε***_DNF_* | noise vector | Randomly distributed values between 0 and 1 |
|  | *β* | sigmoid slope | 4 |
|  | *u_0_* | sigmoid threshold | 0 |
|  | *w_excite_* | self-excitation | 0.9 |
|  | *w_inhibit_* | surround inhibition | 1.25 |
|  | *σ_DNF_* | weight kernel Gaussian width | 2 |
